# Pan-genome and transcriptome analyses provide insights into genomic variation and differential gene expression profiles related to disease resistance and fatty acid biosynthesis in eastern black walnut (*Juglans nigra*)

**DOI:** 10.1093/hr/uhad015

**Published:** 2023-02-01

**Authors:** Huijuan Zhou, Feng Yan, Fan Hao, Hang Ye, Ming Yue, Keith Woeste, Peng Zhao, Shuoxin Zhang

**Affiliations:** College of Forestry, Northwest A&F University, Yangling, Shaanxi 712100, China; Key Laboratory of Resource Biology and Biotechnology in Western China, Ministry of Education, College of Life Sciences, Northwest University, Xi’an, Shaanxi 710069, China; Xi’an Botanical Garden of Shaanxi Province, Xi’an, Shaanxi 710061, China; Key Laboratory of Resource Biology and Biotechnology in Western China, Ministry of Education, College of Life Sciences, Northwest University, Xi’an, Shaanxi 710069, China; College of Forestry, Northwest A&F University, Yangling, Shaanxi 712100, China; Key Laboratory of Resource Biology and Biotechnology in Western China, Ministry of Education, College of Life Sciences, Northwest University, Xi’an, Shaanxi 710069, China; Key Laboratory of Resource Biology and Biotechnology in Western China, Ministry of Education, College of Life Sciences, Northwest University, Xi’an, Shaanxi 710069, China; Xi’an Botanical Garden of Shaanxi Province, Xi’an, Shaanxi 710061, China; USDA Forest Service Hardwood Tree Improvement and Regeneration Center (HTIRC), Department of Forestry and Natural Resources, Purdue University, 715 West State Street, West Lafayette, Indiana, 47907, USA; Key Laboratory of Resource Biology and Biotechnology in Western China, Ministry of Education, College of Life Sciences, Northwest University, Xi’an, Shaanxi 710069, China; College of Forestry, Northwest A&F University, Yangling, Shaanxi 712100, China

## Abstract

Walnut (*Juglans*) species are used as nut crops worldwide. Eastern black walnut (EBW, *Juglans nigra*), a diploid, horticultural important woody species is native to much of eastern North America*.* Although it is highly valued for its wood and nut, there are few resources for understanding EBW genetics. Here, we present a high-quality genome assembly of *J. nigra* based on Illumina, Pacbio, and Hi-C technologies. The genome size was 540.8 Mb, with a scaffold N50 size of 35.1 Mb, and 99.0% of the assembly was anchored to 16 chromosomes. Using this genome as a reference, the resequencing of 74 accessions revealed the effective population size of *J. nigra* declined during the glacial maximum. A single whole-genome duplication event was identified in the *J. nigra* genome. Large syntenic blocks among *J. nigra*, *Juglans regia*, and *Juglans microcarpa* predominated, but inversions of more than 600 kb were identified. By comparing the EBW genome with those of *J. regia* and *J. microcarpa*, we detected InDel sizes of 34.9 Mb in *J. regia* and 18.3 Mb in *J. microcarpa*, respectively. Transcriptomic analysis of differentially expressed genes identified five presumed *NBS-LRR* (*NUCLEOTIDE BINDING SITE-LEUCINE-RICH REPEAT*) genes were upregulated during the development of walnut husks and shells compared to developing embryos. We also identified candidate genes with essential roles in seed oil synthesis, including *FAD* (*FATTY ACID DESATURASE*) and *OLE* (*OLEOSIN*). Our work advances the understanding of fatty acid bioaccumulation and disease resistance in nut crops, and also provides an essential resource for conducting genomics-enabled breeding in walnut.

## Introduction

Eastern black walnut (*J. nigra* L., 2n = 32) is an ecologically important, wind pollinated, fine hardwood [1, 2], with an extensive geographic range in the central and eastern USA, from New England to Texas [2–4]. It belongs to the section *Rhysocaryon*, which includes other North American taxa and several Central and South American species [2, 5–7]. Nearly all members of the genus *Juglans* are valued for their timber; eastern black walnut (EBW) is among the most commercially valuable hardwood timber horticulture species in continental USA [8–10]. Eastern black walnut is also an important industrial crop for its nut shells [11]. There is a market for EBW embryos too, which are like those of Persian walnut (*Juglans regia* L.), i.e. high in protein, total monounsaturated fatty acids, and oleic acid. Like Persian walnut embryos, EBW embryos are used in as food products, although the flavor of EBW embryos is distinctive [11–13]. EBW embryos contain phenolics, high levelsof phytosterols, unsaturated fatty acids, and tocopherols, compounds with benefits for human health and nutrition [12, 13]. *J. nigra* is considered a candidate medicinal tree species becauseof its flavonoids [14, 15]. Much of the character of EBW woodand embryos is determined by their high content of phenoliccompounds [10, 12–14]. These contribute to its rich, darkly colored heartwood, highly tannic fruits, and characteristically aromatic vegetation [10, 14].

Eastern black walnut (*J. nigra*) is considered a tertiary germplasm resource for the improvement of Persian walnut (*J. regia*)*,* themost commercially important species in this genus [15–18]. *J. nigra* expresses traits such as high-quality heartwood fortimber, cold-hardiness, vegetative vigor [3, 11, 17], and diseasetolerance/resistance, that are useful for Persian walnut rootstock breeding [15, 19, 20]. Although pests and diseases rarely limit the growth or utilization of EBW, it is locally challenged by biotic stresses from diseases and pests [3, 4, 20], and abiotic stresses such as salinity, drought, and cold [4, 21]. Genes associated with disease and stress responses are important to walnut researchers and breeders [22, 23]; these include *NBS-LRR* (*NUCLEOTIDE BINDING SITE-LEUCINE-RICH REPEAT*) genes, which regulate responses to biotic stresses in many plants [24–26]. Specifically, *NBS-LRR* were associated with the severity of anthracnose in walnut [26, 27].

The biology, morphology, genetics, and breeding of *J. nigra* have been described [1, 2, 6, 7, 27]. To meet demand for improved EBW varieties, the development of genotypic tools for breeding and the study of evolution, population genetics, and diversity of *J. nigra* began in the 1980s [11], and expanded considerably over the past decade [14, 27]. These tools include the publication of marker systems such as allozymes and microsatellites [2, 28, 29], population genetic studies [30], preliminary genomes [5, 31], and various specialized transcriptomes [23, 32, 33]. EBW is an ideal candidate for purposeful breeding using marker-assisted selection (MAS), functional genes, and genome data. Present and potential uses for these tools include but are not limited to breeding, seed orchard management, cultivar identification, studies of diversity and adaptation, and chain of custody verification for international timber markets [8, 11]. Although a high-quality chromosome-level genome assembly is an essential genetic resource for improving horticultural traits and breeding in woody crops [34–36], genomic resources for breeding in eastern black walnut remain largely undeveloped. Recently published genetic and genomic data have accelerated Persian walnut (*J. regia*) improvement [16, 37–39], and comparative genomic studies among walnut (*Juglans*) species offer the potential to verify genetic factors related to a range of phenotypes and development.

Here, we report a high-quality, chromosome-level *de novo* genome assembly of *J. nigra* (genotype NWAFU168). The whole genome of *J. nigra* was generated using a combination of short reads (Illumina Hiseq) and long read (PacBio) sequencing data, and high-throughput chromatin conformation capture (Hi-C) mapping platforms. We identified EBW-specific expansion of gene families and analysed variation in genome structure and population structure via the genome resequencing of 74 section *Rhysocaryon* accessions. We described aspects of the evolution, size, and structure of the *NBS-LRR* gene family in *J. nigra*. We produced transcriptome expression profiles of flowers and fruits (husks, shells, and embryos) to reveal EBW lipid metabolism. We further identified structural genes associated with fruiting and their potential regulators using time-ordered comparative transcriptome analyses. The genome sequence of EBW reported here is essential for the advanced breeding of *J. nigra*, for comprehending the response of EBW to biotic and abiotic stresses, and for exploring its potential as a rootstock for *J. regia*.

## Results

### *De novo* assembly high-quality improved genome and gene annotation of *J. nigra*

**Figure 1:**
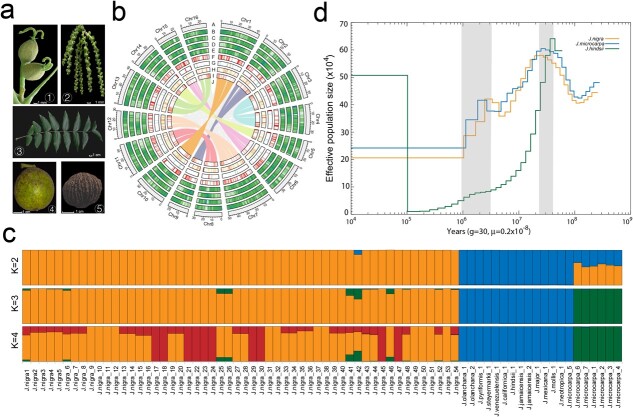
Morphology, genome map, population structure, and demographic history of *Juglans nigra*. **a** morphology of *J. nigra* female flowers (1), male flowers (catkins) (2), leaf (3), mature fruit (4), and nut (5). **b** Circos map of the *J. nigra* genome assembly: A, number of chromosomes; B, gene density; C, LTR density; D, *Gypsy*; E, *Copia*; F, miRNA; G, rRNA; H, snRNA; I, tRNA; J, syntenic relationships among chromosomes. **c** Model-based population structure analysis of 74 section *Rhysocaryon* accessions (*K* from 2 to 4). **d** PSMC estimates of the effective population sizes of *J. nigra* (yellow), *Juglans microcarpa* (blue), and *Juglans hindsii* (green); grey shading indicates Riss-Würm interglacial period (13–11 Mya) and the glacial maximum 3–1 Mya.

To achieve a high-quality *J. nigra* genome assembly, we combined short reads Illumina Hiseq sequence data, long read PacBio sequencing technology sequence data, and Hi-C mapping sequence data. We sequenced total of ~23.6 Gb Illumina sequencing clean reads, ~55 Gb Hi-C clean reads, and ~ 15.9 Gb PacBio long reads equivalent to ~43.6×, ~100.8×, and ~28.7× genome coverage, respectively (Table S1, see online supplementary material). Using the Hi-C mapping technology, the scaffolds were further anchored onto sixteen chromosomes (for *J. nigra* as for all *Juglans*, 2n = 2X = 32) that covered ~98.0% of the assembled sequences (Fig. S1; Tables S2 and S3, see online supplementary material). The final genome assembly was 540.8 Mb with a contig N50 of 21.7 Mb, which is close to the genome size (553.8 Mb) estimated by means of 17-K-mer statistics (Table 1; Figs S1 and S2, Table S1, see online supplementary material). Self-alignment analysis revealed duplicated regions within and between chromosomes (Fig. 1; Fig. S1, see online supplementary material). The lengths of the 16 assembled chromosomes of *J. nigra* ranged from 22 101 821 bp to 50 134 109 bp, with an average length of 33 963 507 bp (Table S3, see online supplementary material). We compared our *J. nigra* genome assembly (genotype NWAFU168) with the previously *J. nigra* assemblies: ‘Sparrow’ [28] and ASM291648 v2 [5] (Table S3 and S4, see online supplementary material). The lengths of the 16 chromosomes of the previous *J. nigra* ‘Sparrow’ assembly [31] ranged from 17 790 123 bp (chr16) to 43 016 657 bp (chr1), with an average length of 30 054 810 bp. The assembly of ASM291648 v2 [5] ranged from 19 540 967 bp (pseudoChr15) to 52 028 492 bp (pseudoChr7), with an average length of 32 865 598 bp (Table S3, see online supplementary material). Our assembly of *J. nigra* were highly syntenic with the previous two *J. nigra* assemblies [5, 31], although the length of each chromosome differed for each of the three *J. nigra* genome assemblies (Fig. S3, see online supplementary material), and we observed inversions between the *J. nigra* assembly NWAFU168 and other two assemblies (Fig. S3, see online supplementary material).

**Table 1:** Statistical summary of the assembly and annotation of eastern black walnut (*Juglans nigra*) genotype NWAFU168.

Genomic feature	Value
Total genome size (bp)	540 839 626
N50 contig length (Mb)	21.7
N50 scaffold length (Mb)	35.1
GC content (%)	36.5
Size of retrotransposons (Mb)	229.1
Size of DNA transposons (Mb)	17.2
Size of total repeat sequences (Mb)	272.4
Protein-coding gene number (*n*)	29 506
Mean coding sequence length (bp)	1192
Mean exons per gene (*n*)	5.15
Mean exon length (bp)	23.4
Mean intron length (bp)	803.4
BUSCO completeness (%)	99.0

We used four analytical methods to estimate the completeness of *J. nigra* genome assembly. First, we verified a total of 1614 BUSCO (Benchmarking Universal Single-Copy Orthologs) groups, 1598 (99.0%) complete BUSCOs, 8 fragmented BUSCOs, 129 duplicated BUSCOs, and 1469 single copy BUSCOs in the NWAFU168 assembly (Table S5, see online supplementary material). Second, based on the CEGMA (Core Eukaryotic Genes Mapping Approach), 243 core eukaryotic genes (98.0%) were verified in NWAFU168 assembly. Third, we aligned the Illumina short read data (23.6 Gb) to our completed genome assembly, and 99.2% of the clean reads were mapped. Finally, we mapped re-sequencing short read data from 54 EBW accessions to the EBW genome NWAFU168 assembly (sample details in Table S6, see online supplementary material), and the mean mapping rate of whole-genome resequencing reads from 54 *J. nigra* individuals was 97.2% (Table S6, see online supplementary material). These four-assessments validated the quality of the NWAFU168 assembly showed that the EBW genome assembly is of good quality in both genic and intergenic regions.

We annotated a total of 29 506 protein-coding gene models from the *J. nigra* NWAFU168 genome, with a mean CDS (coding sequence) length of 1193 bp and a mean of five exons per gene (Table 1). Among 29 506 predicted genes, there were 29 151 (96.3%) genes annotated in the Nr (non-redundant) database, 28 644 (94.7%) genes were annotated in InterPro (Protein sequence analysis and classification), 23 106 (76.4%) genes were annotated in KEGG (Kyoto Encyclopedia of Genes and Genomes), and 23 065 (76.2%) genes were annotated in Pfam (The Pfam protein families database) (Table S7, see online supplementary material). We also annotated a large component of non-coding RNA including rRNA (ribosomal RNA), tRNA (transfer RNA), snRNA (small nuclear RNA), and miRNA (microRNA) (Table S8, see online supplementary material). Total of 5431 rRNA, 1036 tRNA, 855 snRNA, and 372 miRNAs were identified (Table S8, see online supplementary material).

### Genomic variation and demographic history of *J. nigra*.

Our whole-genome resequencing focused on 54 *J. nigra* and 20 other sect. *Rhysocaryon* species (so-called ‘black walnuts’). Resequencing resulted in 6.8 Gb Illumina clean reads, with a mean depth of 19.9× and an average coverage of 92.1% of the assembled *J. nigra* genome (Table S6, see online supplementary material). This dataset allowed us to identify 10 676 835 single nucleotide polymorphisms (SNPs) and 3 523 538 insertions and deletions (InDels) in the germplasm collection (Table S6, see online supplementary material). During mapping, we identified 3 142 846 heterozygous SNPs, showing that the nucleotide completeness rate of the EBW genome assembly was 99.99%. As mentioned above, we found an average mapping rate among the *J. nigra* samples of 97.2%, whereas for the other sect. *Rhysocaryon* samples the average mapping rate was 97.7% (Table S6, see online supplementary material). The high mapping rates of other sect. *Rhysocaryon* species onto the *J. nigra* reference reflect their shared ancestry and strong genetic affinity. High quality assemblies from one member of sect. *Rhysocaryon* will likely prove extremely useful for other section members that have not yet been assembled.

Population genetic analysis divided three genetic clusters within *Rhysocaryon*: *J. nigra*, *Juglans microcarpa*, and other black walnut species (Fig. 1c), however, we considered two genetic clusters to be the best representation based on cross-validation (CV) errors (Fig. S4, see online supplementary material). Pairwise genetic distances between re-sequenced samples were envisioned by averages of an NJ (neighbor-joining) phylogenetic tree, which also indicated three distinct clades (Fig. S5a, see online supplementary material). Principal component analysis (PCA) further separated accessions of *J. californica*, *J. jamaicensis*, *J. major*, *Juglans hindsii*, *J. mollis*, *J. olanchana*, *Juniperus pyriformis*, *Juglans neotropica*, *Jasarum steyermarkii*, and *J. venezuelensis* more clearly than the phylogenetic tree (Fig. S5b, see online supplementary material). We identified signals of gene introgression from the *J. microcarpa* clade into *J. nigra* populations (Fig. 1c).

To detect the demographic history of black walnut, we employed the mismatch distribution curve, which showed a single peak Poisson distribution, indicating that EBW populations went through a demographic expansion in the past (Fig. S6, see online supplementary material). Using whole-genome resequencing data, we performed the PSMC method to test the dynamic history effective population size (Ne) of *J. nigra* populations, *J. hindsii,* and *J. microcarpa* during the climatic oscillations (Fig. 1d). We revealed a divergence and decline in Ne for all three black walnut species associated with the well-known Riss-Würm interglacial period (13–11 million years ago, Mya), a decline from which *J. hindsii* did not recover. The population sizes of all three species appear to have stabilized briefly during the glacial maximum 3–1 Mya in the Northern Hemisphere (Fig. 1d), although they subsequently declined again.

### Transposable elements

**Figure 2:**
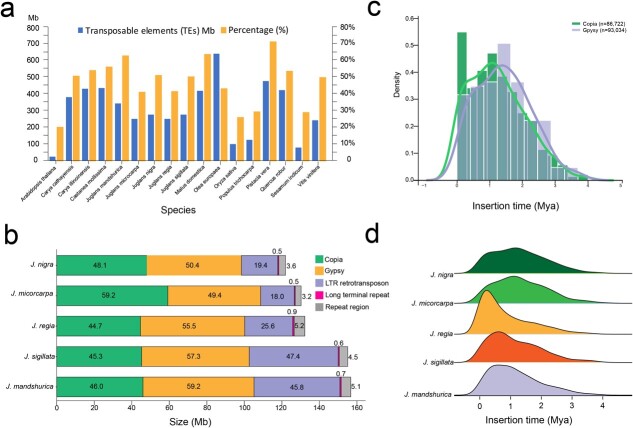
Analysis of transposable elements (TEs) in *Juglans nigra*. **a** The size and percentage of the entire genome occupied by TEs in *J. nigra* and in 16 other plant species. **b** Genomic constituents in *J. nigra* in comparison with those in *Juglans microcarpa*, *Juglans regia*, *J. sigillata*, and *Juglans mandshurica*. All five constituents, especially *Gypsy,* were abundant in walnut genomes. **c** Insertion breaks of *Gypsy* and *Copia* elements in *J. nigra*. **d** Temporal patterns and insertion time of LTR-RT in *J. nigra* as compared to those in *J. microcarpa*, *J. regia*, *J. sigillata*, and *J. mandshurica*.

Analysis of repeat elements showed that LTR-RTs (long terminal repeat retrotransposons), including *Copia*, *Gypsy*, and LTR retrotransposon, were the dominant transposable elements (TEs) in the EBW genome. These comprised 87.3% of the annotated TE content, and 44.6% of the assembled *J. nigra* genome (Fig. 2a). Cross-genome comparisons with *J. regia*, *J. sigillata*, *J. microcarpa*, and *Juglans mandshurica* showed that the loss or gain of LTR-RTs, especially LTR retrotransposon elements, contributed the most to differences in size between the genome of EBW and its congeners (Fig. 2a). EBW (*J. nigra*) had ~48.1 Mb more LTR-RTs than *J. sigillata*, *J. regia*, and *J. mandshurica*, but not *J. microcarpa*. EBW had ~50.4 Mb less of *Gypsy* elements DNA than did *J. regia* (55.5 Mb). The *J. nigra* genome had less LTR retrotransposon element sequence (~18.4 Mb) than did *J. sigillata* and *J. mandshurica*, and this difference explained much of the difference in genome size between *J. nigra* and *J. mandshurica* (Fig. 2b). By comparing our *J. nigra* assembly with other walnut genome assemblies (*J. regia, J. mandshurica*, and *J. microcarpa*) [40–42] (Fig. 2), we identified a large number of TE super families in EBW (Fig. 2). The role of TE expansion and contraction in the evolution of *Juglans* needs additional study. TE bursts might be associated with speciation in tertiary relict walnuts, especially in cases where hybridization may have played a role in speciation, such as in the origin of *Juglans cinerea*.

A distinctly bimodal distribution was found for the insertion bursts of intact *Copia* and *Gypsy* retroelements in black walnut, while a single peak was observed for *J. regia*, *J. microcarpa*, *J. sigillata*, and *J. mandshurica* (Fig. 2c). EBW and *J. microcarpa* showed a high percentage of old LTR-RT insertions, with the expansion burst appearing around 1.5 million years ago (Mya). A second peak in *J. nigra* occurred approximately 0.2 Mya; this peak was also seen in *J. regia* (Fig. 2c). We found earlier bursts of *Copia* elements than *Gypsy* in EBW (Fig. 2c). Apparently, there were large-scale bursts of *Copia* and *Gypsy* in *J. nigra* around 1.5–1.3 Mya. The *J. nigra* genome shows evidence of two earlier insertion events compared with *J. regia*, *J. microcarpa*, *J. mandshurica*, and *J. sigillata* (Fig. 2d).

### Whole-genome duplication

We investigated the evidence for and consequences of whole-genome duplication events (WGD) in the EBW (*J. nigra*) genome by comparing three walnut genomes (*J. nigra*, *J. microcarpa*, and *J. regia*) [40, 42], and the genome of grape (*Vitis vinifera*) (Figs S7–S10, see online supplementary material). Paralogous relationships among the sixteen EBW (*J. nigra*, NWAFU168) chromosomes (Fig. S7, see online supplementary material), jointly containing 5329 pairs of paralogous genes in all blocks of *J. nigra* genome (Fig. 1; Fig. S8, see online supplementary material). Both paralogous relationships (Fig. S7, see online supplementary material) and dot-plot alignments (Fig. S8, see online supplementary material) showed eight main duplication sub genomes (chromosome pairs as follows: Chr1 and Chr10, Chr2 and Chr9, Chr3 and Chr4, Chr5 and Chr14, Chr6 and Chr15, Chr7 and Chr12, Chr8 and Chr11, and Chr13 and Chr16) within the assembled eastern black walnut (*J. nigra*, NWAFU168) chromosomes (Figs S7 and S8, see online supplementary material). We observed similar inversions, collinear genes, and segmental duplications among the *Juglans* genomes, but there were considerable differences between the *J. nigra* and *V. vinifera* chromosomes (Fig. S9, see online supplementary material). The peak of synonymous nucleotide substitution (Ks) was ~0.4 and ~1.3 for orthologous gene pairs within the black walnut assembly, indicating that *J. nigra* evolved through two polyploidy events (Fig. S10, see online supplementary material). Compared to the *V. vinifera* genome, all five walnut species had one Ks peak of ~0.9 for orthologous gene pairs, which represented divergence between genes duplicated by the γWGT (whole-genome triplication). Because the peaks of Ks had different values for the orthologous gene pairs between each of the two walnut genomes, the genomes reflect species differentiation (Fig. S10, see online supplementary material).

### Comparative genomic analysis and gene family evolution of *J. nigra*

**Figure 3:**
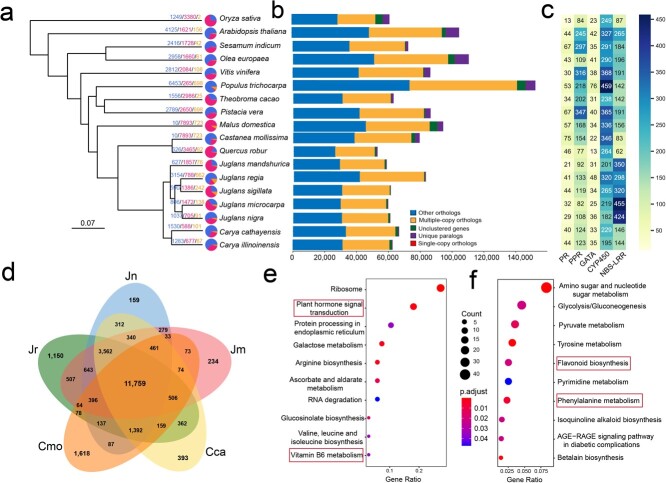
*Juglans nigra* genome evolution. **a** Expanded, contracted, and rapidly evolving gene families in 18 species; pie charts on each branch of the phylogenetic tree indicate the proportion of genes showing expansion (blue), loss (red), and rapid evolution (orange), the numbers near the nodes shows number of expanded or contracted gene families. The font color indicates the number of genes gained (blue), lost (red), and rapidly evolving (orange). **b** The proportion of multicopy genes, unique paralogues, single-copy orthologues, other paralogues, and unclustered genes among 18 species; rows correspond to species as listed in panel **a**. **c** The number of *PR*, *PPR*, *GATA*, *CYP450*, and *NBS-LRR* genes among the species shown in panel **a**; colors are intended as a visual guide to gene number. **d** Venn diagram of the gene space of five woody perennial species in Fagales (Jn = *J. nigra*, Jr = *Juglans regia*, Jm = *Juglans microcarpa*, Cmo = *Castanea mollissima*, and Cca = *Carya cathayensis*). Regions of overlap indicate shared orthologs. **e** KEGG analysis of gene families that were expanded, contracted, and rapidly evolving in the *J. nigra* genome. **e** KEGG analysis of 159 unique paralogs of *J. nigra*.

To understand how the EBW genome evolved, we compared eighteen representative genomes, including several from the walnut family (Juglandaceae) (Fig. 3). The comparison included 18 angiosperm species for which high-quality genomes are available (for details, see Table S9, see online supplementary material). We identified 2160 single-copy genes of black walnut with orthologs in all eighteen angiosperm species (Fig. 3a). Compared with the other plant species, in EBW 1037 gene families were expanded, 705 gene families were contracted, and 91 gene families were rapidly evolving (Fig. 3a; Table S9, see online supplementary material). The rapidly expanding gene families were associated with plant hormone signal transduction, vitamin B6 metabolism, pathogenesis-related protein (*PR*), pentatricopeptide repeat (*PPR*), Glutamyl-tRNA amidotransferase, subunit A (*GATA*), cytochrome P450 (*CYP450*), and *NBS-LRR* gene families (Fig. 3e; Table S9, see online supplementary material). We then performed a genome-wide counting of five pathogenesis-related gene families (*PR*, *PPR*, *GATA*, *CYP450*, and *NBS-LRR*) among eighteen genomes (Fig. 3c). The *NBS-LRR* genes, which expanded in *J. nigra* and *J. microcarpa*, were contracted in *J. regia* (Fig. 3c). We found that the five Fagales species, i.e. *J. nigra*, *J. mandshurica*, *J. regia*, *Castanea mollissima*, and *Carya cathayensis*, shared 11 759 genes, whereas 159 orthogroups were specific to *J. nigra* (Fig. 3d). KEGG analysis of these 159 unique genes of *J. nigra* showed that they regulate phenylpropanoid metabolism, flavonoid biosynthesis, and amino sugar and nucleotide sugar biosynthesis (Fig. 3f).

### Genome-wide variation within *Juglans* genomes

**Figure 4:**
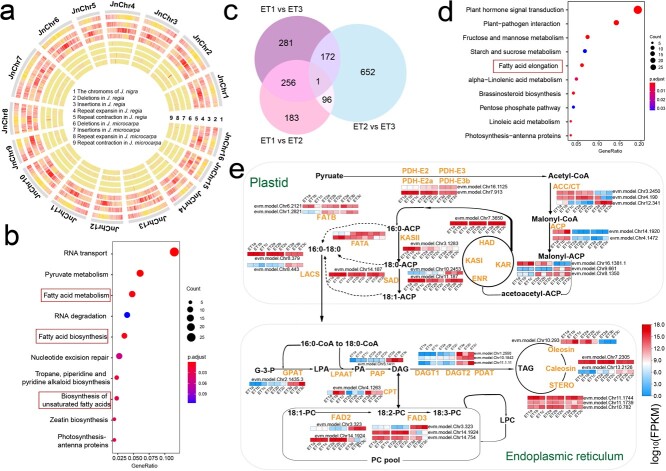
Comparative analysis of the genomes of *Juglans nigra*, *Juglans regia*, and *Juglans microcarpa* and lipid biosynthesis in *J. nigra*. **a** Circos map of InDels and repeats for each *J. nigra* chromosome versus the *J. regia* and *J. microcarpa* assemblies: (1) number of chromosomes; the (2) deletions, (3) insertions, (4) repeat expansion, and (5) repeat contraction detected between *J. nigra* genome and *J. regia* genome; the (6) deletions, (7) insertions, (8) repeat expansion, and (9) repeat contraction detected between *J. nigra*, *J. regia*, and *J. microcarypa*. **b** KEGG analysis of InDels between the first development stage (ET1) and third development stage (ET3) of *J. nigra* embryo development. **c** Venn diagram of the DEGs among three development stages of black walnut embryo (80 DAF, 111 DAF, and 140 DAF). **d** KEGG analysis of DEGs (281 genes) between the first development stage (ET1) and third development stage (ET3) of *J. nigra* embryo development. **e** Transcriptional profiles of lipid biosynthetic genes in developing *J. nigra* embryos. The expression levels of lipid biosynthesis genes in the developing *J. nigra* embryo based on transcriptome data. The nine colored panes in each horizontal row represent three stages with three biological replications (80 DAF, 111 DAF, and 140 DAF). Blue to red colored squares indicate log_10_FPKM values.

To reveal and examine genome-wide variations within walnut (*Juglans*) genomes we employed chromosome to chromosome comparisons between the *J. nigra*, *Juglans macrocarpa*, *J. regia* and *J. mandshurica* (Fig. 4a; Figs S11 and S12, see online supplementary material). We identified large syntenic blocks between the *J. nigra* genome (sect. *Rhysocaryon*) and its relatives *J. regia* (sect. *Dioscaryon*) and *J. mandshurica* (sect. *Cardiocaryon*) (Fig. S11, see online supplementary material). Syntenic blocks were also identified between the genomes of *J. nigra* and *Carya illinoinensis*, an important nut crop (pecan) and a more distant relative within the Juglandaceae (Fig. S12, see online supplementary material). Synteny between EBW and *J. mandshurica* chromosomes 2, 3, 4, 5, 6, 10, 11, 12, 14, 15, and 16 was especially strong. Within *Juglans*, we identified larger syntenic blocks between EBW (*J. nigra*) and *J. regia* than between EBW and *J. mandshurica* (Fig. S12, see online supplementary material). Interestingly, synteny between EBW and *C. illinoinensis* (chromosomes 1, 7, 8, 13, and 16) was generally focused on different chromosomes than the synteny within congeners (Fig. S12, see online supplementary material). Moreover, we found that synteny between EBW and *J. mandshurica* chromosomes 1, 7, 8, 9, 13, and 15 had larger inversions within chromosome compared to the synteny between EBW and *J. regia* (Fig. S12, see online supplementary material). These synteny differences might reflect differences in the evolution of chromosomes among the three walnut species (Fig. 4a and b; Fig. S12, see online supplementary material).

We described the InDels of the EBW genome by comparison between *J. regia* and *J. microcarpa* (Fig. 4a; Fig. S13, see online supplementary material); these totaled 34.9 Mb (6.5%) in *J. regia* and 18.3 Mb in *J. microcarpa* (Fig. 4a; Figs S13–S15, see online supplementary material). InDels and repeats in *J. regia* were obviously greater in number than in *J. nigra* or its close relative, *J. microcarpa* (Fig. 4a; Figs S13–S15, see online supplementary material). A comparison of *J. nigra* and Persian walnut showed large numbers of InDels on the chromosomes 3, 4, 7, 11, and 13; InDels between *J. nigra* and *J. microcarpa* were also concentrated on chromosomes 7 and 13 (Fig. S13, see online supplementary material). The mean density of InDels detected between the genomes of EBW and *J. regia* was 12.5 (InDels bp^−1^ chromosome^−1^), whereas the mean density of InDels between the genomes of EBW and *J. microcarpa* was 3.4. The most common insertions and deletions ranged in size from 50 to 500 bp (Fig. S14, see online supplementary material). InDels >10 kb were uncommon across the *J. nigra* genome (Fig. S14, see online supplementary material). In total, 2180/136047 InDels were more than 5 kb in *J. regia* (about 1.6% of all InDels), but only 0.9% of the total in *J. microcarpa* (312/34457). A total of 2180 genic InDels >5 kb was verified in *J. nigra* compared to *J. regia*. Enrichment analysis of genes absent in *J. nigra* as compared with *J. regia* showed a significant (− log10 *P* > 10) depletion of genes related to metabolism of fatty acids and pentose phosphate (Fig. 4b). Annotation of these 2180 genes showed a strong association with RNA transport, pyruvate metabolism, biosynthesis of unsaturated fatty acid, and fatty acid metabolism and biosynthesis (Fig. 4b). We also identified 312 genic InDels >5 kb in *J. nigra* as compared to *J. microcarpa* (Fig. S15, see online supplementary material). Annotation of these 312 genes showed a strong association with glycolysis/gluconeogenesis, alpha-linolenic acid metabolism, pentose phosphate pathway, and fatty acid degradation. These genetic differences might be expected to contribute to differences in oil content and composition among the three species (Fig. 4a and b; Fig. S15, see online supplementary material).

### Transcriptomics profiling of *J. nigra* embryos in lipid biosynthesis

To detect the profiles of transcriptome associating with the development of EBW fruits, we constructed 33 RNA-seq libraries; 27 were from developing walnut husks, developing shells, and developing embryos, and the remaining were from other six tissues (Table S10, see online supplementary material). We obtained 207.8 Gb clean data, with an average of 6 296 194 063 clean reads of each accession and 97.5% Q20 ratio per sample (Table S10, see online supplementary material). The *J. nigra* embryo (nut kernel) is rich in polyunsaturated fatty acids. To further detect and profile the transcriptome associated with the development of EBW oils, we constructed nine libraries from developing walnut embryos (Table S10, see online supplementary material). Using a pairwise comparison analysis, we identified a total of 1641 DEGs (differential expressed genes) between samples at three collection times (Fig. 4c). Annotation of these 525 overlapped genes that were expressed in more than one sample (overlap regions of Fig. 4c.) revealed a strong association with plant−pathogen interaction, plant hormone signal transduction, starch and sucrose metabolism, fatty acid degradation, and fructose and mannose metabolism (Fig. 4d). To elucidate the mechanisms of black walnut embryo development, we focused on the trends of DEGs related to lipid biosynthesis (Fig. 4e). We identified 312 unigenes expressed in the embryo samples related to oil (lipid) biosynthesis, including 165 unigenes for fatty acid biosynthesis (in plastids), and 157 for fatty acid biosynthesis in the endoplasmic reticulum (Fig. 4e). Some genes related to fatty acid synthesis were highly abundant at all three development stages of EBW embryo, including *PDH* [*PYRUVATE DEHYDROGENASE*, evm.model.Chr7.913, mean FPKM (fragments per kilobase of exon model per million mapped fragments) value = 194.9], *HAD* (*3-HYDROXYACYL-ACP DEHYDRATASE*, evm.model.Chr7.3650, mean FPKM value = 68.2), *FATB* (*FATTY ACYL-ACP THIOESTERASES B*, evm.model.Chr6.2121, mean FPKM value = 77.8), and *SAD* (*STEAROYL-ACP DESATURASE*, evm.model.Chr14.187, mean FPKM value = 28.9) in plastid, and the members of the *OLE* (*OLEOSIN*) family (evm.model.Chr7.2305, mean FPKM value = 2654.4), *ENR* (*ENOYL-ACYL CARRIER PROTEIN REDUCTASE*, evm.model.Chr11.187, mean FPKM value = 651.5), and *FAD* (*FATTY ACID DESATURASE*, evm.model.Chr14.1924, mean FPKM value = 641.2; evm.model.Chr14.754, mean FPKM value = 313.0) in endoplasmic reticulum (Fig. 4e). Two genes were expressed more highly in the first stage *ACEE* (*PYRUVATE DEHYDROGENASE E1 COMPONENT*, evm.model.Chr7.913) and *KAS I* (*3-KETOACYL-ACP SYNTHASE I*, evm.model.Chr4.1237), while genes that were expressed more highly in the third stages of EBW embryo development included *OLE* (*OLEOSIN*, evm.model.Chr13.2126), *HAD* (*3-HYDROXYACYL-ACP DEHYDRATASE*, evm.model.Chr7.3650), *ENR* (*ENOYL-ACYL CARRIER PROTEIN REDUCTASE*, evm.model.Chr7.3467), and *ABCD* (*ABC TRANSPORTER SUBFAMILY D*, evm.model.Chr2.1144 and evm.model.Chr14.1280) (Fig. 4e). We identified 1137 gene models related to lipid synthesis that were differentially expressed based on FPKM values among the three embryo time stages (ET1-ET3) (Fig. 4e). The *J. nigra* genome assembly contained orthologs to all the essential lipid biosynthesis genes in EBW (Fig. 4e).

### *NBS-LRR* members in *J. nigra*

**Figure 5:**
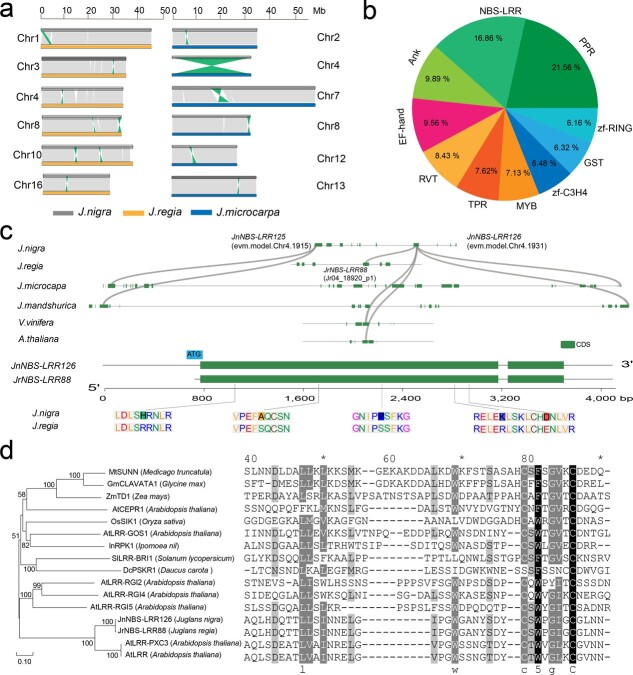
Collinearity analysis of resistance-related genes and comparative analysis of the *NBS-LRR* genes in *Juglans nigra* and *Juglans regia*. **a** Genome collinearity between *J. nigra*, *J. regia*, and *Juglans microcarpa*. Regions of collinearity smaller than 100 kb are filtered out. The green and purple lines indicate syntenic blocks more than 600 kb in length. **b** The KEGG enrichment analysis of InDels of *J. nigra* and *J. regia*. **c** Gene structure and collinearity of *JnNBS-LRR126* and homologous *NBS-LRR* genes in *J. regia*, *J. microcarpa*, *Juglans mandshurica*, *Vitis vinifera*, and *Arabidopsis thaliana*. **d** The phylogenetic tree and protein sequences of *JnNBS-LRR126* and other *NBS-LRR* genes.

In general, the *J. nigra*, *J. regia*, and *J. microcarpa* genomes were highly syntenic along each chromosome (Fig. S16, see online supplementary material), but we observed frequent intrachromosomal rearrangements, especially inversions. For example, the comparison of EBW (*J. nigra*) with Persian walnut (*J. regia*) demonstrated eight inversions of more than 600 kb on chromosomes 1, 3, 4, 8, 10, and 16 (Fig. 5a; Fig. S16, see online supplementary material). We characterized the structure variations (SVs) in the assembly of EBW (*J. nigra*) compared to Persian walnut (Fig. 5a). Twenty-five large SVs (>5 kb) containing translocations and inversions were found between the EBW and Persian walnut genomes, with the lengths ranging from 40 242 bp to 49 410 bp (Fig. 5a; Fig. S15, see online supplementary material). Gene function annotation within the inverted regions indicated that many of those genes were correlated to disease resistance (*R* genes), including *NBS-LRR*, myb DNA-binding domain (*MYB*), and pentatricopeptide repeat (*PPR*) gene families (Fig. 5b).

To gain further insight into the important disease resistance gene family *NBS-LRR* in *J. nigra*, we compared our newly assembled *J. nigra* genome to the *J. regia* genome by performing an evolution and expression profile of *NBS-LRR* genes. By screening candidate protein domains, we identified 430 genes putatively encoding NBS-LRR proteins in *J. nigra* and 298 genes in *J. regia*, a significantly different number (Figs S17 and S18, see online supplementary material). To further explore the disease resistance genes of *J. nigra*, we used sequence homology to identify candidate genes in *J. nigra* based on gene function studies in *J. regia*. *JnNBS-LRR126* was located at chromosome 4 in the *J. nigra* assembly. The chromosomal localizations of *NBS-LRR* genes on the chromosome 4 in the two walnut genomes showed that *JnNBS-LRR126* had high collinearity with *JrNBS-LRR88* (Fig. 5c). The gene structure of *JnNBS-LRR126* in *J. nigra*, *J. regia*, *J. microcarpa*, *J. mandshurica*, *V. vinifera*, and *Arabidopsis thaliana* assemblies (Fig. 5c) showed that *J. nigra*, *J. mandshurica*, and *J. microcarpa* have two highly conserved homologs of this *NBS-LRR* gene (*JnNBS-LRR125* and *JnNBS-LRR126*), while *J. regia*, *V. vinifera*, and *A. thaliana* contain one homolog, i.e. *JrNBS-LRR88*, located within a 1.2 Mb region on chromosome 4 (Fig. 5c). The conserved first CDS sequences of *JnNBS-LRR126* and *JrNBS-LRR88* had five protein variations (Fig. 5d). A maximum likelihood phylogenetic and protein sequence alignment analysis showed that JnNBS-LRR126 and JrNBS-LRR88 were most closely related to AtLRR-PXC3 (O22938.1 and NP_181713.1) in *A. thaliana* (Fig. 5d; Table S11, see online supplementary material).

**Figure 6:**
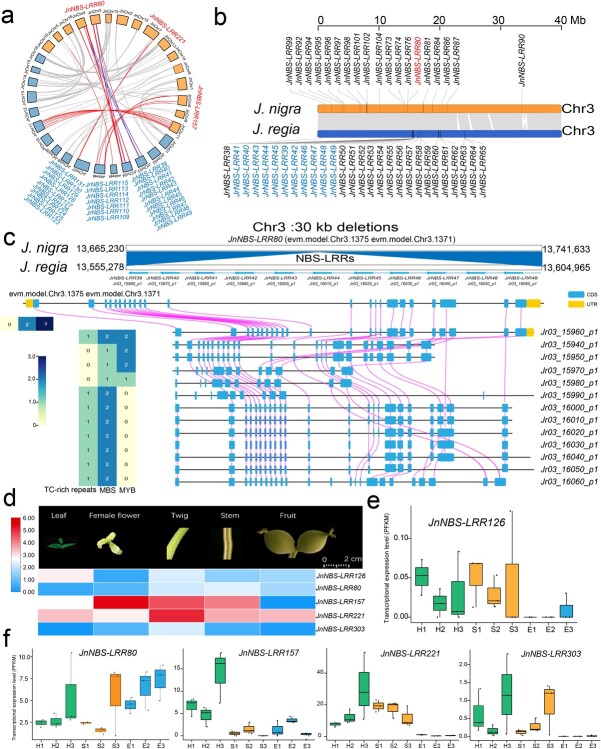
*NBS-LRR* gene cluster collinearity and expression profiles in *Juglans nigra* and *Juglans regia*. **a** Genome-wide synteny analysis of *NBS-LRR* genes for *J. nigra* and *J. regia*. Orthologous and paralogous *NBS-LRR* genes were mapped onto the chromosomes and then linked. Red lines and blue lines indicate orthologous and paralogous gene pairs in chromosomes 1, 3, 4, 6, 7, 8, and 11 in *J. nigra* and *J. regia*. Grey lines indicate orthologous and paralogous gene pairs in other remaining chromosomes. **b***NBS-LRR* gene chromosomal distribution and gene collinearity of chromosome 3 in *J. nigra* and *J. regia*. The large-scale deletion (0.02 Mb) on chromosome 3 of the *J. nigra* genome contained one disease resistance gene *JnNBS-LRR80* (red), while the *J. regia* genome contains eleven *NBS-LRR* genes (blue) in the same region. **c** Gene structure and synteny analysis of *JnNBS-LRR126* and 11 *J. regia NBS-LRR* genes on chromosome3. Sequence comparison between *J. nigra* chromosome 3 and *J. regia* chromosome 3: the upper figure shows, in grey, extensive regions of collinearity >100 kb; the middle figure shows the region from 13 665 230 bp to 136 741 633 bp on the chromosome 3 of *J. nigra* that includes 11 genes (*NBS-LRR*) deleted in *J. regia*; the bottom figure shows *NBS-LRR* gene collinearity on the chromosome 3 between *J. regia* (from 13.54 Mb to 13.83 Mb) and *J. nigra* (from 13.94 Mb to 14.02 Mb). **e**–**f** The morphology of five tissue/organs (leaf, female flower, twig, stem, and fruit) and expression heat map of *JnNBS-LRR126*, *JnNBS-LRR80*, *JnNBS-LRR157*, *JnNBS-LRR221*, and *JnNBS-LRR303* in those five tissues. Expression levels of those five *NBS-LRR* genes during three development stages of *J. nigra* husks, shell, and embryo. (H = husk; S = shell; E = embryo; for details of samples, see Table S10).

The *NBS-LRR* genes on chromosomes 3, 4, 6, 7, 8, and 11 of *J. nigra* showed a linear similarity with *NBS-LRR* genes on the same chromosomes in *J. regia* (Fig. 6a; Fig. S19, see online supplementary material). In general, the *NBS-LRR* gene members were distributed on both ends of chromosomes 1, 2, 7, 10, and 16 in the *J. regia* genome, but their distribution was more uniform in *J. nigra* (Fig. 6a; Figs S17 and S18, see online supplementary material). Chromosomes 3, 4, 6, 7, 8, and 11 of these two walnut species genomes had the highest collinearity (Fig. 6a; Fig. S19, see online supplementary material). In *J. nigra NBS-LRR* genes, *JnNBS-LRR80* (Chr3), *JnNBS-LRR157* (Chr6), *JnNBS-LRR221* (Chr7), and *JnNBS-LRR303* (Chr11) showed high homology with *NBS-LRR* gene clusters on the same-numbered chromosomes of *J. regia* (Fig. 6a and b; Fig. S19, see online supplementary material). For example, the single *J. nigra NBS-LR*R gene *JnNBS-LRR80* on chromosome 3 corresponds to a cluster of 11 homologs in *J. regia*: *JrNBS-LRR39, JrNBS-LRR40, JrNBS-LRR41, JrNBS-LRR42, JrNBS-LRR43, JrNBS-LRR44, JrNBS-LRR45, JrNBS-LRR46, JrNBS-LRR47, JrNBS-LRR48*, and *JrNBS-LRR49* (Fig. 6a–c). Expansion at this locus in *J. regia* produced large-scale variation (0.02 Mb) on chromosome 3 (Fig. 6c). The gene structure of *JnNBS-LRR80* and eleven *JrNBS-LRRs* showed that the CDS had collinearity with each other (Fig. 6c). The domains and gene structure of these 12 *NBS-LRR* genes were conserved, but the gene length was considerably different (Fig. 6c). Although we characterized a total of 430 *NBS-LRR* genes in *J. nigra* (Figs S17 and S18, see online supplementary material), all the *NBS-LRR* genes on chromosome 3 of *J. nigra* showed a low percentage of similarity with genes on the *J. regia* chromosome 3 (Fig. 6c; Table S12, see online supplementary material). We determined that the promoter of *JnNBS-LRR80* contains two NBS and three MYB elements, while the 11 *NBS-LRR* genes in the related NBS gene cluster in *J. regia* contain fewer NBS and MYB elements (Fig. 6c). By examining the promoters of these 12 *NBS-LRR* genes, we found that the *J. nigra* versions generally contained more NBS and MYB elements than their homologs in *J. regia*. This finding highlights the potential role of promoter element content in the regulation of disease resistance in *Juglans* (Fig. 6c; Fig. S20, see online supplementary material).

### *NBS-LRR* gene expression in black walnut

We measured the expression profiles of five candidate *NBS-LRR* genes from *J. nigra*: *JnNBS-LRR126*, *JnNBS-LRR80*, *JnNBS-LRR157*, *JnNBS-LRR221*, and *JnNBS-LRR303* in developing walnut husk/shells, leaves, twigs, stems, and female flowers of *J. nigra* (Fig. 6). Among these five tissues, *JnNBS-LRR221* was expressed highly in leaves, twigs, stems, and fruit, while *JnNBS-LRR157* was expressed highly in female flowers, twigs, and stems (Fig. 6d). *JnNBS-LRR126* was expressed higher in leaves than in any other tissue evaluated (Fig. 6d).

To detect and profile the transcriptome associated with *JnNBS-LRR126, JnNBS-LRR80, JnNBS-LRR157, JnNBS-LRR221,* and *JnNBS-LRR303*, we constructed 27 libraries from developing walnut husks, developing shells, and developing embryos (Fig. 6e–f; sample details in Table S10, see online supplementary material). We obtained 189.5 Gb clean data, with an average of 7 017 490 111 clean reads of each accession and 97.52% Q20 ratio per sample (Table S10, see online supplementary material). In general, *JnNBS-LRR126*, *JnNBS-LRR80*, *JnNBS-LRR157*, *JnNBS-LRR221*, and *JnNBS-LRR303* expressed significantly more highly levels in developing walnut husks than developing shells and embryos (Fig. S21, see online supplementary material; Fig. 6e–f). Based on the mean FPKM value of transcriptome, the expression of *JnNBS-LRR157*, *JnNBS-LRR221*, and *JnNBS-LRR303* was greatest at the third collection time point in late September (Fig. 6e–f). *JnNBS-LRR221* expression increased during husk development but decreased during shell development (Fig. 6e–f). *JnNBS-LRR80* expression generally increased across all developmental time points in all three tissues, except for a slight dip in expression in the endocarp (Fig. 6f).

## Discussion

The genus *Juglans* includes around 23 species that produce large, oily nuts, although there is an international commercial market for the nuts of *J. regia* only [37–40, 43]. *J. nigra* (EBW) is best known as a timber species, although humans have also selected nut varieties of EBW for over 100 years [2, 7, 30, 44], and a nut breeding program for EBW is supported by the University of Missouri [7, 30]. *J. nigra* belongs to the section *Rhysocaryon* [1], which includes nearly all other New World *Juglans*, e.g. little walnut (*J. microcarpa* Berl.), *J. hindsii* (Jeps.) Rehder, *J. californica* S. Wats., *J. major* (Torr. ex Sitsgr.), *J. mollis* Engelm. ex Hemsl., and *J. steyermarkii* Mann., etc. [1]. The *J. nigra* genome we present in this manuscript will be relevant for sect. *Rhysocaryon* species in particular. *J. nigra* is especially valued among *Juglans* for its timber, disease resistance, and cold tolerance [21, 26]. Disease tolerance/resistance within *J. nigra* and other members of section *Rhysocaryon* make them attractive as rootstocks for *J. regia* [24, 27, 44, 45]. The potential role of the *NBS-LRR* genes in walnut (*Juglans*) disease resistance has been reported [21, 27, 46, 47].

We constructed a high-quality, chromosome-level genome assembly of *J. nigra* that contains 29 506 protein-coding genes (Table S6, see online supplementary material). We assembled the *J. nigra* genome to be 540.8 Mb, similar in size to other *Juglans* species, such as *J. regia* (606 Mb) (Chandler 2.0), *J. mandshurica* (540 Mb) [41], and *J. sigillata* (548 Mb) [48], but smaller than some other Juglandaceae (Fagales) species, such as *Carya cathayensis* (750 Mb) [49], *C. illinoinensis* (‘Oaxaca’, 650 Mb) [34], and *C. mollissima* (785.5 Mb) [50] in the Fagaceae. For our *J. nigra* genome assembly, the value of contig N50 was improved 88.6-fold and 9.0-fold compared to the previously available *J. nigra* genome assemblies (Table S4, see online supplementary material) [5, 31]. The final *J. nigra* genome size is smaller than the previous genome version (620 Mb, ‘Sparrow’ versus 540 Mb, NWAFU168), while larger than the previous genome version (532 Mb, ASM291648 v2 versus 540 Mb, NWAFU168) (Table S4, see online supplementary material). The results of resequencing 74 section *Rhysocaryon* accessions indicated that our assembly will be a good reference for the population genetic analysis of EBW and other related species (Fig. 1; Table S6, see online supplementary material).

Based on a comparison with 18 representative species, many of the expanded, contracted, and rapidly evolution gene families in the EBW (*J. nigra*) genome were associated with plant hormone signal transduction, vitamin B6 metabolism, pathogenesis (e.g. *PPR*, the pentatricopeptide repeat family), and *CYP450* (Fig. 3). A total of 159 unique paralogues were enriched in *J. nigra*, these were associated with amino sugar metabolism, nucleotide sugar metabolism, flavonoid biosynthesis, and phenylpropanoid metabolism, and their role in the biology of *J. nigra* remains to be determined (Fig. 3e).

Our genome assembly allowed us to compare the locations of InDels, SNPs, inversions, and structure variations (SVs) among *Juglans* species (Fig. 4; Fig. S15, see online supplementary material). The prediction of the function of genes containing InDels, inversions, and SVs indicated that some are related to the disease resistance and biosynthesis of fatty acids, including members of *NBS-LRR*, *MYB*, glutathione s-transferase (*GST*), and *PPR* gene families (Fig. 3). Some disease resistance related genes such as *NBS-LRR* in *J. nigra* reflected species-specific variation, probably as a result of selection under speciation and adaption. On chromosome 3 of the *J. regia* genome we found a 0.02 Mb insertion that includes 11 *NBS-LRR* disease resistance genes. In the context of walnut evolution, diversification, and domestication, large structural variants could have led to changes in disease tolerance/resistance.

NBS-LRR proteins are among the most important classes of resistance genes (*R* genes) in plants [51]. Comparison of our *J. nigra* assembly with the genome of its congener *J. regia* revealed the extent and location of genome evolution within *Juglans*. For example, although the two species have a similar number of genes and similar genome sizes [39], we determined that the number of *NBS-LRR* gene family members in *J. nigra* was 1.5× greater than the number in *J. regia*. The distribution of *NBS-LRR* members in the *J. nigra* genome was relatively even as compared to *J. regia*, in which *NBS-LRR* genes were clustered. In some cases, a single *NBS-LRR* gene of *J. nigra* showed high similarity and collinearity with an entire cluster of *NBS-LRR* genes of *J. regia*. The expansion and clustering of genes related to resistance has also been observed in the *WAK* (*WALL ASSOCIATED KINASE WAK/WAKL*) gene family in Persian walnut and *J. mandshurica* [52], and it is believed to be related to maintenance of key functions, neo-functionalization, and in some cases, as in the species-specific duplication and clusters of *NBS-LRR*, evolution in response to selection pressure [52–54].

The identification of *NBS-LRR* genes identification in eastern black walnut is the first step toward characterizing their role in resistance to biotic stresses. For example, a comprehensive analysis of RNA sequencing data indicated that *NBS-LRR* genes are activated in plant-microbe interactions in several walnut (*J. regia*) tissues [22]. *J. nigra* and *J. regia* are both susceptible to anthracnose caused by *Gnomonia leptostyla* [55]. A candidate genetic marker derived from an *NBS-LRR* gene but of no known function was associated with anthracnose susceptibility in both *J. nigra* and *J. regia* genotypes [22]. Recently, it was shown that *JrWRKY21* was significantly upregulated upon *C. gloeosporioides* infection in *J. regia* [56]. An and Yang (2014) identified a large number of what they called resistance gene analogs that contained domains associated with *NBS-LRR* genes that were associated with resistance to the pathogen *Colletotrichum gloeosporioides* in *J. regia* [57]. In tomato (*Solanum lycopersicum*), it was reported that long non-coding RNAs (LncRNA) regulate the expression of *NBS-LRR* genes[58]. In *J. regia*, there is evidence that LncRNAs are involved in the regulation of resistance to *Colletotrichum gloeosporioides* based on treatments of AR (anthracnose-resistant) and AS (anthracnose-susceptible) walnut fruits [46, 47]. Comparative genomics analysis will be providing good tools to reveal *R* genes in walnut species [25, 41]. Of particular importance in EBW are genes activated in response to infection by the fungi *Geosmithia morbida*, *Diaporthe capsica*, *Fusarium spp.*, and *Nectria galligena*, which are associated with a diseases of *J. nigra* [59–62].

The oil content of most *Juglans* species is poorly characterized, presenting an opportunity for comparative metabolomics to contribute to our understanding of seed oil production [52, 63]. Seed oil content and composition have not (yet) been important targets for improvement in *J. nigra*, although they are in other oil seed crops, including *J. regia* [63]. The *SAD*, *FAD2*, and *FAD3* were upregulated highly abundantly expressed in the *J. regia* embryo [63], which indicates that these candidate genes might play an essential role in the polyunsaturated fatty acids biosynthesis in *J. regia* [64]. Other genes [*FUS3* (*B3 DOMAIN-CONTAINING TRANSCRIPTION FACTOR*), *ABI3* (*ABSCISIC ACID INSENSITIVE 3*), *LEC2* (*LEAFY COTYLEDON 2*), and *WRI1* (*WRINKLED 1*) transcription factors] may be involved in the regulation of lipid biosynthesis in *J. mandshurica* [52]. We found that transcripts for *LEA*, *FAD*, *OLE*, *LTP* (*LIPID TRANSFER PROTEINS*), *ERD* (*EMBRYOGENIC CELL PROTEIN*), and *SSP* (*SEED STORAGE PROTEIN*) were expressed at extremely high levels during three EBW embryo development stages, indicating their possible role in black walnut oil synthesis and/or embryo pattern formation and maturation. The third development stage of shells was associated with oil synthesis and maturity of the walnut fruit. These data lay the genomic foundation for breeders to improve the oil contents and to modify the fatty acid compositions in *Juglan*s.

In summary, we assembled a high-quality genome assembly of *J. nigra*, a valuable and attractive multi-use species. Our work will not only provide a genome resource for understanding the genomic basis of genomic variations in oil synthesis and disease resistance in *J. nigra* and other members of the *Juglans*, it is expected to accelerate genome-enabled breeding of walnut (*Juglans*) and other Juglandaceae for traits related to nut quality and disease resistance.

## Materials and methods

### Sample collection, DNA extraction, library construction, and genome sequencing

In 2021 we collected young, heathy leaves from a single individual of *J. nigra* (genotype NWAFU168), growing in the orchard of Northwest A&F University, Yangling, Shaanxi, China (altitude: 500 m, 34°17′N, 108°05′E). Total genomic DNA of NWAFU168 was prepared from the fresh leaf samples using a kit (TIANGEN, Beijing, China). A DNA library (150 bp) was analysed using short read sequencing with the Illumina HiSeq X Ten platform (San Diego, CA, USA) for short read sequencing. For long read sequencing, genomic DNA was extracted using DNA Template Prep Kit 3.0 (Pacific Biosciences, USA). PacBio SMRTbell long-read (20 kb) libraries were constructed and then sequenced on the PacBio Sequel platform using P6-C4 chemistry (Novogene, Beijing). For the chromosome-level genome Hi-C sequencing, we prepared a Hi-C library that was sequenced on the Illumina NovaSeq6000 platform (San Diego, CA, USA). After filtering out the low-quality reads, we obtained ~23.6 Gb (43.6×) of Illumina clean short-reads, 15.9 Gb (28.7×) of long-reads, and ~ 55 Gb (100.8×) of the Hi-C clean reads (Table S1, see online supplementary material).

### Genome assembly and assessment

The Illumina raw reads were evaluated with SOAPnuke v1.5.6 to filter adapters or low-quality data [65]. The genome assembly size was estimated by means of 17-K-mer statistics (Fig. S2, see online supplementary material) [66]. *De novo* assembly of *J. nigra* was performed with Falcon v1.87 [67]. The sequencing data from PacBio and Hi-C platform were mapped to assembled scaffolds using BWA-aln [68]. Based on Hi-C data, scaffolds were anchored to 16 pseudomolecules using LACHESIS software [69] resulting in an N50 of approximately 27 Mb (Table 1). The interaction heatmap of *J. nigra*’s 16 chromosomes was produced using HiC-pro software [70].

We evaluated the quality and completeness of the *J. nigra* genome assembly using the BUSCO v3.0.2 (bench marking universal single-copy orthologs) [71]. Secondly, we also evaluated genome assembly by mapping Illumina DNA short reads using BWA-MEM (Burrows-Wheeler Alignment-Maximal Exact Match) [72]. We further evaluated the genome by mapping transcripts using the software GMAP [73]. Finally, we evaluated genome completeness by mapping Illumina resequencing reads from 74 individuals (Table S8, see online supplementary material) to the *J. nigra* genome assembly using BWA [72]. *J. nigra* genome assembly features were visualized using Circos [74].

### Annotation of protein-coding genes

Genome annotation and the prediction of protein-coding genes were undertaken using transcriptomic data, homology-based annotation, and *de novo* prediction methods [41]. RNA sequences from six tissues (dry stigma, mature leaves, bark from stems, immature fruit, new shoots, and stem; Table S10, see online supplementary material) were used to annotate genes using the software AUGUSTUS [75]. For homology-based annotation, gene structure was predicted for protein-coding genes with reference to five species or species hybrids (*J. regia*, *C. illinoinensis*, *J. regia* × *J. microcarpa*, *C. mollissima*, and *A. thaliana*) using Exonerate v2.2.0 [76]. The final annotation of the protein-coding genes was processed using MAKER2 [77] and AUGUSTUS [75] that combined the results from homologous protein-coding gene mapping, transcriptome sequence mapping, PacBio mapping, and whole-genome resequencing mapping [41]. We evaluated the final functional annotation of protein-coding genes based on six databases, including SwissProt (Swiss Institute of Bioinformatics and Protein Information Resource) [78], Nr (non-redundant) [79], KEGG (Kyoto Encyclopedia of Genes and Genomes) [80], InterPro (protein sequence analysis and classification) [81], GO (gene ontology) [82], and Pfam (the Pfam protein families database) [83] databases (details in Table S7, see online supplementary material).

### Repeat annotation

To identify transposable elements (TEs), the *J. nigra* genome sequence was searched and blasted against databases using Repbase v.20.05 [84], RepeatMasker v.4.0.7 [85], Tandem Repeats Finder (TRF) v4.09 [86], PILER [87], and RepeatProteinMask v4.0.7 [85] with default parameters. The LTR-RTs (long terminal repeat retrotransposons) were identified in *J. nigra* genome and selected genomes using LTRharvest v.1.5.10 [88]. We mapped the translated sequences to the two superfamilies *Copia* and *Gypsy*, which were aligning by the software MAFFT with default parameters (https://mafft.cbrc.jp/alignment/software/).

### Genome features analysis

The genome circle plot combined the distribution patterns of various genomic features including the density of gene, LTR, Gypsy, Copia, miRNA, rRNA, snRNA, tRNA, and syntenic relationships among chromosomes. The syntenic relationships within the species *J. nigra* was obtained using the MCSCANX software [89], in which the Evalue was set to 1E-3 and the number of BlastHits was set to 10 [90]. The pair of two chromosomes containing the greatest collinearity was considered homologous chromosomes. The density of the features was calculated using BEDTOOLS with 1 Mb sliding windows [91]. The genome circle plot was visualized using the ‘advanced ciros’ function in TBtools software [92].

### Genomic variation analysis

To investigate the population-level features of the *J. nigra* genome, we compared whole-genome resequencing data from 74 sect. *Rhysocaryon* accessions with the *J. nigra* reference we produced. These 74 genomes included 54 *J. nigra* and other 20 (sect. *Rhysocaryon*) species (so-called ‘black walnuts’); 30 of these genomes were published in previous studies (Table S8, see online supplementary material) [44]. All genome re-sequencing reads from the 74 accessions (~20× genomic coverage, for individual information details see Table S8, see online supplementary material) were mapped to the EBW (*J. nigra*) reference genome assembly NWAFU168 using the BWA [41, 68] with default parameters. We identified SNPs (single nucleotide polymorphisms) and InDels (insertions and deletions) using the program GATK pipeline (Genome Analysis Toolkit) [93]. Firstly, the sequencing data was filtered using the software FASTP with default parameters. After raw data filtering, the clean reads were aligned to the reference genome *J. nigra* NWAFU168 using the BWA-MEM with the parameters ‘−T 20 −k 30’ [68]. Then, we named the alignments (response to each sample) as SAM files using the software SAMTOOLS [94]. We compared the sequencing depth and coverage using the *J. nigra* NWAFU168 assembly. We then called the duplication reads, SNPs and InDels using PICARD and GATK with the joint calling strategy [93]. Specifically, we obtained all samples’ genomic variants GVCF files, which are combing with the module ‘CombineGVCFs’. We detected the final genomic variations of SNPs and InDelS via the ‘GenotypeGVCFs’ and ‘HaplotypeCaller’ modules. The filter criterias of SNPs and InDels as follows: (i) we filtered the SNPs near an Indel within the length of 5 bp and adjacent Indels within 10 bp using a perl script vcfutils.pl package in the program BCFTOOLS [94]; (ii) the variations filtered using GATK with the ‘VariantFiltration’ module [93]; and (iii) by removing the variations, we performed subsequent pruning with missing genotype rates >20% or minor allele frequencies (MAF) <0.05 using the software PLINK [95].

### Population genomic analysis

All genome re-sequencing reads from *J. nigra* accessions (~20× genomic coverage) were mapped to the *J. nigra* NWAFU168 reference genome assembly using BWA [94] with default parameters. We performed a NJ (neighbor-joining) phylogenetic tree of 74 individuals based on a total of 92 560 670 SNPs using software MEGA [96]. We performed genetic cluster analysis using Bayesian clustering software ADMIXTURE v1.30 [97], with the *K* value from 2 to 15 corresponded to the different numbers of genetic clusters. The *K* value was estimated using the lowest rate of change in cross-validation error [98]. Principal component analysis (PCA) was evaluated with EIGENSOFT v7.2.1 [99]. Demographic history and Ne (effective population size) were estimated using a PSMC (pairwise sequentially Markovian coalescence) model v0.6.4-r49 [5]. We estimated the changes of the effective population size over time for three black walnut species: *J. nigra*, *J. microcarpa,* and *J. hindsii*. The PSMC (pairwise sequentially Markovian coalescence) model v0.6.4-r49 [5] was used to determine the demographic history and effective population size. First, binary alignment map (.bam) was converted to the Fastq format using the SAMTOOLS and BCFTOOLS to create pseudo diploid heterozygous genomes [94]. Population size history was estimated using the PSMC package with the parameters –p ‘4 + 25 × 2 + 4 + 6’, in which the mutation rate was set to 2.09E-8 per site per year, and the generation time was set to 30 (years) [5].

### Comparative genomics

To understand how the EBW genome evolved, we reconstructed the genomes of the walnut family (Juglandaceae) including *Carya* species [*C. illinoinensis* (pecan) and *C. cathayensis*)] [49], Chinese chestnut (*C. mollissima*) [50], Manchurian walnut (*J. mandshurica*) [41], Persian walnut [40], little walnut (*J. microcarpa*) [42], and Iron walnut (*J. sigillata*) [48]. We selected 17 species (*A. thaliana*, *Carya cathayensis, C. illinoinensis*, *C. mollissima*, *J. mandshurica*, *J. microcarpa*, *J. regia*, *Juglans sigillata*, *Malus domestica*, *Olea europaea*, *Oryza sativa*, *Populus trichocarpa*, *Pistacia vera*, *Quercus robur*, *Sesamum indicum*, *Theobroma cacao*, and *V. vinifera*) along with *J. nigra* to perform comparative genome analysis (for details of species information see Table S9, see online supplementary material). Gene families were selected for all-versus-all program BLASTP [90] with an E-value cutoff set to 1e^−5^. We identified gene families using the software OrthoMCL v.2.0.9 [100]. We then summarized the orthogroup gene families from OrthoMCL output using a custom Python script. We constructed a ML (maximum likelihood) phylogenetic tree using the program RAxML v8.2.12 [101] with a total of 2160 single-copy genes. Based on the gene family statistics, we calculated the expansion, contraction, and rapid evolution of gene family numbers using program CAFE [102]. Species divergence times were estimated using PAML v4.5 software and MCMCtree [103]. We drew Venn diagrams for five plant species (*J. nigra*, *J. regia*, *J. microcarpa*, *C. mollissima*, and *Carya cathayensis*) using InteractiVenn (http://www.interactivenn.net/). We performed the synteny analysis of our assembly *J. nigra* NWAFU168 reference genome and three *Juglans* species’ genomes (including *J. regia* [39], *J. mandshurica* [41], *J. microcarya* [42]), as well as *C. illinoinensis* [49], using the software MCScanX [89] and GenomeSyn [104]. We also compared our assembly *J. nigra* NWAFU168 reference genome and the previous *J. nigra* assemblies ‘Sparrow’ [31] and ASM291648 v2 [5] (Tables S3 and S4, see online supplementary material).

### WGD events and synteny analyses

Both synteny and synonymous substitution rate (Ks) were used to investigate the timing of whole-genome duplication (WGD) events in the *J. nigra* genome assembly. We calculated the WGD events using software KaKs_Calculator v2.0 [105]. We applied the Ks values using comparisons of EBW (*J. nigra*), Persian walnut (*J. regia*), and *V. vinifera*. The Ks distributions of orthologues among *J. nigra*, *J. regia*, and *J. mandshurica*, and *J. microcarpa*, and between walnut genomes and *V. vinifera* and walnut genomes were determined using the ggplot2 package [106]. We identified synteny and collinear blocks of genes using MCScanX [89].

### Identification of variation and synteny analysis between *J. nigra*, *J. regia*, and *J. microcarpa*

We identified the InDels (deletion and insertion) and structure variations (SVs) distinguishing *J. nigra* versus Persian walnut (*J. regia*), and EBW (*J. nigra*) versus *J. microcarpa* genome assemblies using the online Assemblytics analysis pipeline [107]. We aligned the *J. nigra* assembly as reference genome against the *J. regia* and *J. microcarpa* genomes using the software Mummer 4.0.0beta2 [108]. All-vs-all BLASTP [90] was performed between *J. nigra* versus Persian walnut (*J. regia*), and *J. nigra* versus *J. microcarpa* genome assemblies with an E-value cutoff setting of 1e^−5^, and synteny blocks containing at least five gene pairs were identified using MCScanX [89] with default parameters. The synteny of chromosome pairs of *J. nigra* versus *J. regia*, and *J. nigra* versus *J. microcarpa* was processed by MCScan [89].

### Transcriptome sequencing and expression analysis.

In this study, we collected six tissues/organs for total RNA extraction and sequencing, i.e. leaves, female flowers, immature fruits, bark, twigs, and stems from a single *J. nigra* genotype NWAFU168 (Table S10, see online supplementary material). We also collected a total of 27 samples from three organs, including walnut husk, shell (developing endocarp), and embryo at three developmental stages 80 days after flowering (DAF), 111 DAF, and 140 DAF with three biological replications. Each of the replications was separately extracted (Table S10, see online supplementary material). All 33 samples (27 fruits plus six tissues) were processed using RNA Library Prep Kit (Beverly, MA, USA), and (paired-end) sequenced using Illumina HiSeq X Ten platform (Illumina, USA) (transcriptome samples details in Table S10, see online supplementary material). All the clean reads were then mapped to the *J. nigra* reference genome using Bowtie2 [109] with default parameters. The numbers of FPKM were used to calculate the gene expression levels [110]. We identified DEGs (differential expressed genes) using DESeq2 [111]. Heatmaps were visualized with the TBtools [91].

### Genome-wide analysis of gene family

Based on the results from CAFE and the plant species’ resistance traits, we selected five rapidly evolving pathogenesis-related gene families, including pathogenesis-related gene (*PR*), PPR repeat (*PPR*), Glutamyl-tRNA amidotransferase, subunit A (*GATA*), cytochrome P450 (*CYP450*), and *NBS-LRR*, and identified their members of gene family in 18 species (*A. thaliana*, *Carya cathayensis, C. illinoinensis*, *C. mollissima*, *J. nigra*, *J. mandshurica*, *J. microcarpa*, *J. regia*, *Juglans sigillata*, *M. domestica*, *O. europaea*, *O. sativa*, *P. trichocarpa*, *P. vera*, *Q. robur*, *S. indicum*, *T. cacao*, and *V. vinifera*). The sequences of putative *PR*, *PPR*, *GATA*, *CYP450*, and *NBS-LRR* genes were used as queries in a BLASTP search against nine protein databases to identify candidate orthologs with parameters E-value <1e-5, identity≥50% and coverage≥50% [90]. We determined the protein domains in the candidate sequences using database of Pfam [83]. The locations of chromosome of *NBS-LRR* genes of *J. nigra* and *J. regia* were determined using TBtools software [94]. We analysed the NJ tree of the *NBS-LRR* members in EBW (*J. nigra*) and Persian walnut (*J. regia*) using MEGA software [96][96]. To detect the *NBS-LRR126* members in *J. nigra* and *J. regia*, we downloaded a total of 20 *PR126* members from NCBI (for details see Fig. S21, see online supplementary material) to construct a phylogenetic tree with MEGA [96]. We conducted the collinearity analysis of *NBS-LRR* genes using the software MCScanX [90]. We performed the prediction of *cis*-acting elements using PlantCARE [112] with the 2000 bp sequences upstream of the *NBS-LRR* genes.

## Acknowledgements

This work was supported by the National Natural Science Foundation of China (32070372, 41471038, and 31800372), the Operating Services of Qinling National Forest Ecosystem Research Station financed by Ministry of Science and Technology of China, Natural Science Basis Research Plan in Shaanxi Province of China (2019JQ-641), Shaanxi Academy of Science Research Funding Project (2019 K-06), and Science Foundation for Distinguished Young Scholars of Shaanxi Province (2023-JC-JQ-22). This work was also supported in part by the United States Department of Agriculture Forest Service. Mention of a trademark, proprietary product, or vendor does not constitute a guarantee or warranty of the product by the US Department of Agriculture and does not imply its approval to the exclusion of other products or vendors that also may be suitable.

## Author contributions

P.Z. and S.Z. conceived and designed the study. F.Y., F.H., and P.Z. collected the samples. F.H., H.Z., and F.Y. took the morphology picture and collected the transcriptome materials of black walnut. H.Z., F.Y., F.H., and P.Z. assembled the genome, and performed gene annotation, gene family, and expression profiles. H.Z., F.Y., M.Y., K.W., H.Y., P.Z., and S.Z. supported the software. F.Y., H.Z., and P.Z. performed the comparative genome analysis. H.Y., H.Z., F.H., and P.Z. performed the population genomic analysis, F.Y. and H.Z. performed the whole-genome duplication and LTRs analysis. H.Z. and P.Z. wrote the draft manuscript and then P.Z., K.W., and S.Z. edited and revised the English writing of this manuscript. All authors contributed to and approved the final manuscript.

## Data availability

The whole genome sequence data including Illumina short reads, Nanopore long reads, Hi-C interaction reads, and transcriptome data have been deposited in the NCBI, under accession numbers: PRJNA801766 (SRR17842367 and SRR17841629). The Illumina whole-genome resequencing data of *J. nigra* in this study have been deposited in China National GeneBank (CNGB) Nucleotide Sequence Archive database under project accession number CNP0001209 (https://db.cngb.org/search/project/CNP0001209/). The transcriptome data of six tissues and organs (leaves, female flowers, immature fruits, mature fruits, bark, and young stem) and husk, shell, and embryo (three developmental stages) have been deposited in the NCBI, under accession numbers: PRJNA799697 (SRR17728328, SRR17728315, SRR17728293, SRR17727056, SRR17715789, SRR17714911, SRR17822893, SRR178417850, RR17817728, SRR17798583, SRR17794307, SRR17777323, SRR17775560, SRR17775247, and SRR17761587).

## Conflict of interest statement

None declared.

## Supplementary Data

Supplementary data is available at *Horticulture Research* online.

## Supplementary Material

Web_Material_uhad015
