# Supplementary material for: Pan-genome and transcriptome analyses provide insights into genomic variation and differential gene expression profiles related to disease resistance and fatty acid biosynthesis in eastern black walnut (Juglans nigra)
Source: Hortic Res. 2023 Feb 1;10(3):uhad015. doi: 10.1093/hr/uhad015 (PMC10031739; doi:10.1093/hr/uhad015)
Supplement: Web_Material_uhad015 [file web_material_uhad015.zip › Supplementary Figures.pdf]

Supplementary Figures

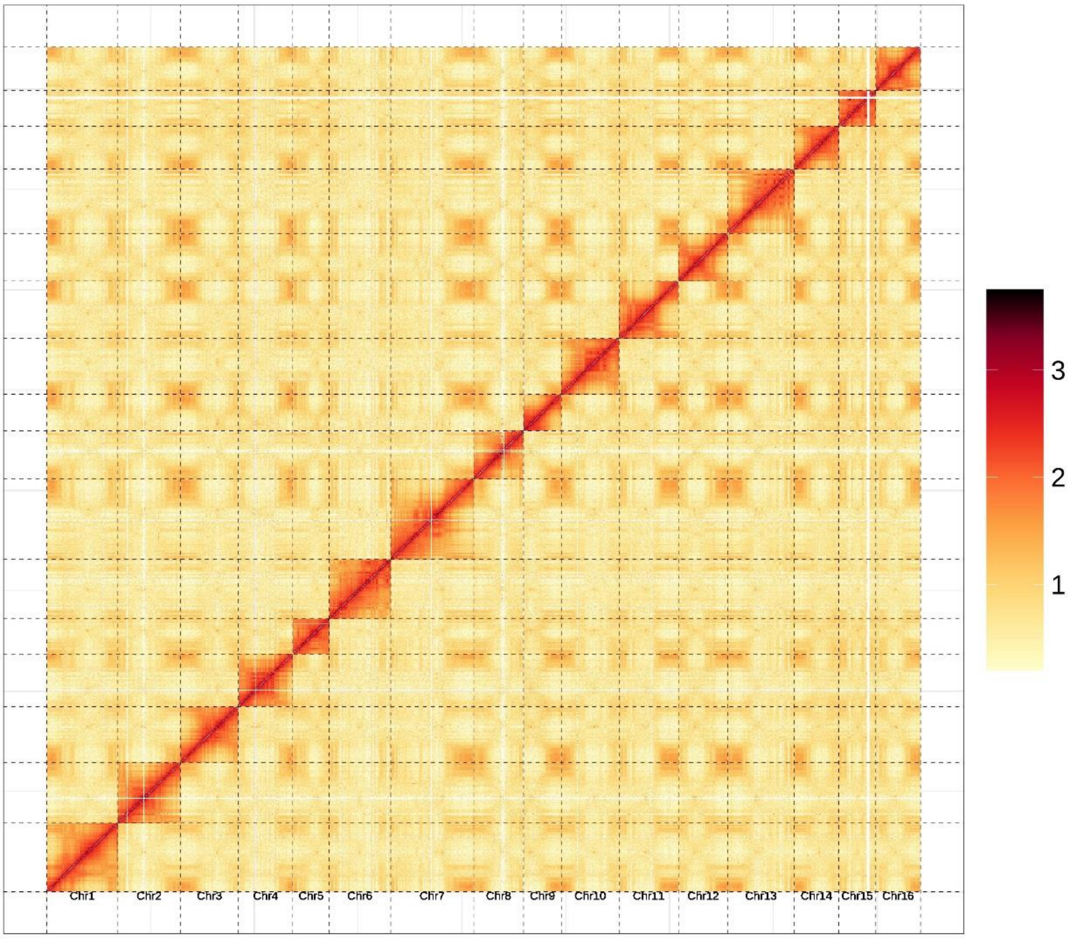

Figure S1 Heatmap of chromosomes of *Juglans nigra* after Hi-C assisted assembly.

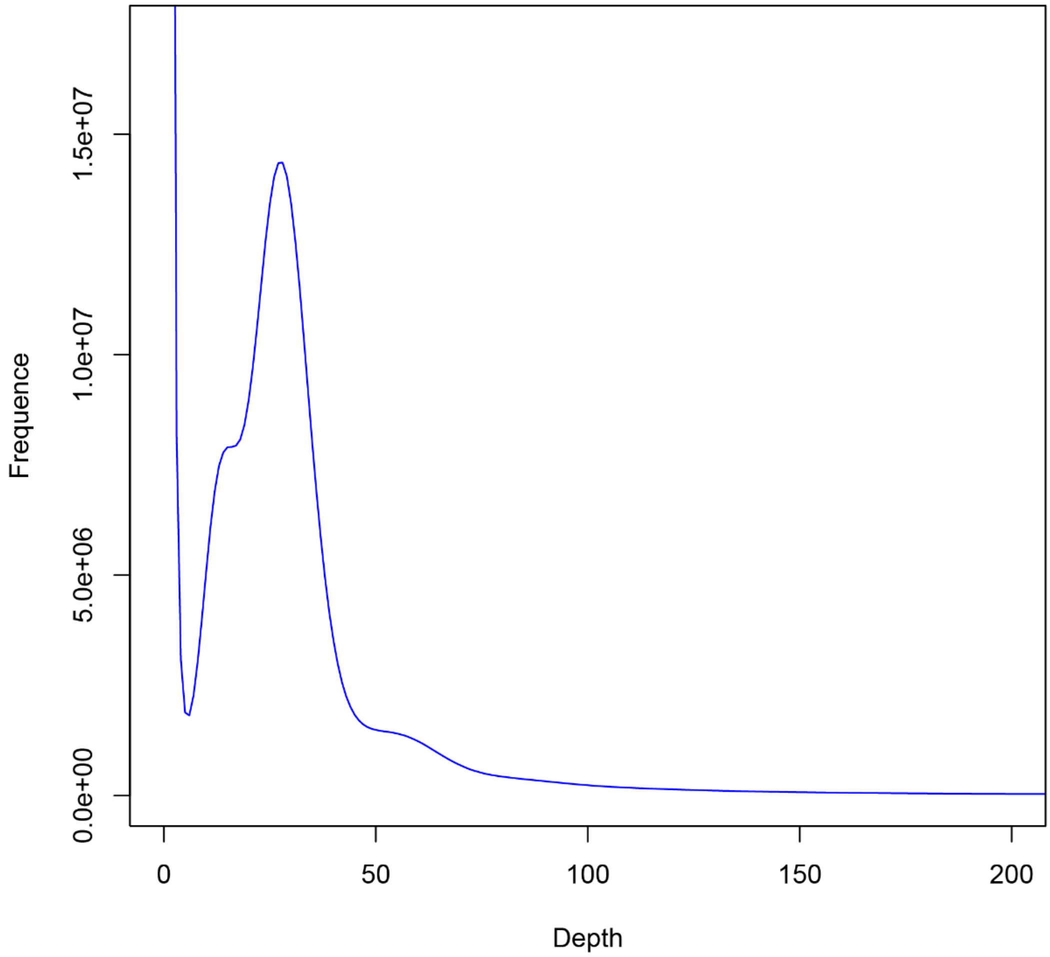

Figure S2 Genome size estimation by 17-K-mer analysis of *Juglans nigra*.

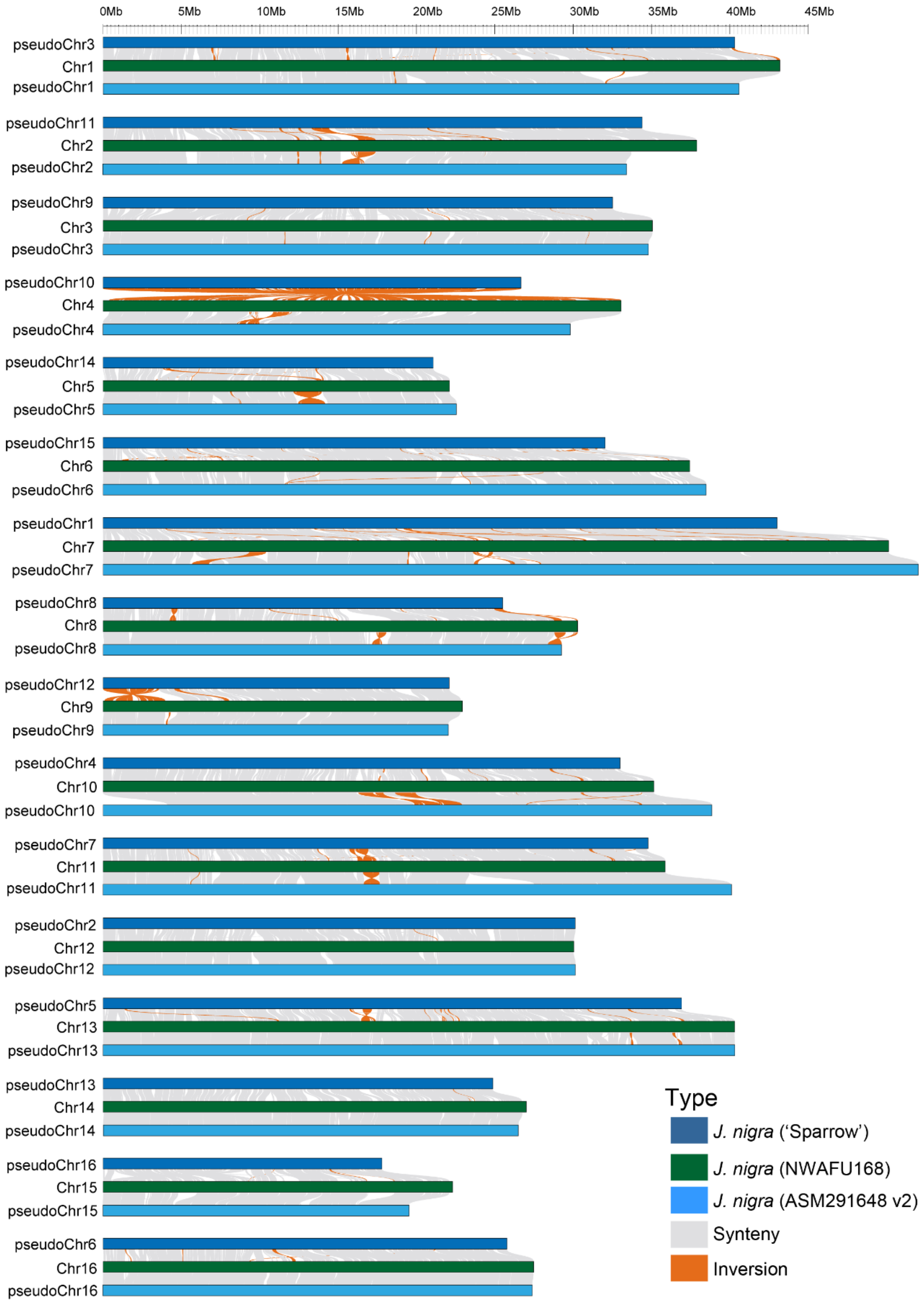

Figure S3 Genome collinearity between three *Juglans nigra* assemblies. NWAFU168 was the *J. nigra* genotype assembled in this study. 'Sparrow' and ASM291648 v2 indicate previous *J. nigra* assemblies. For details of three *J. nigra* assemblies see Supplementary Table S3 and Table S4.

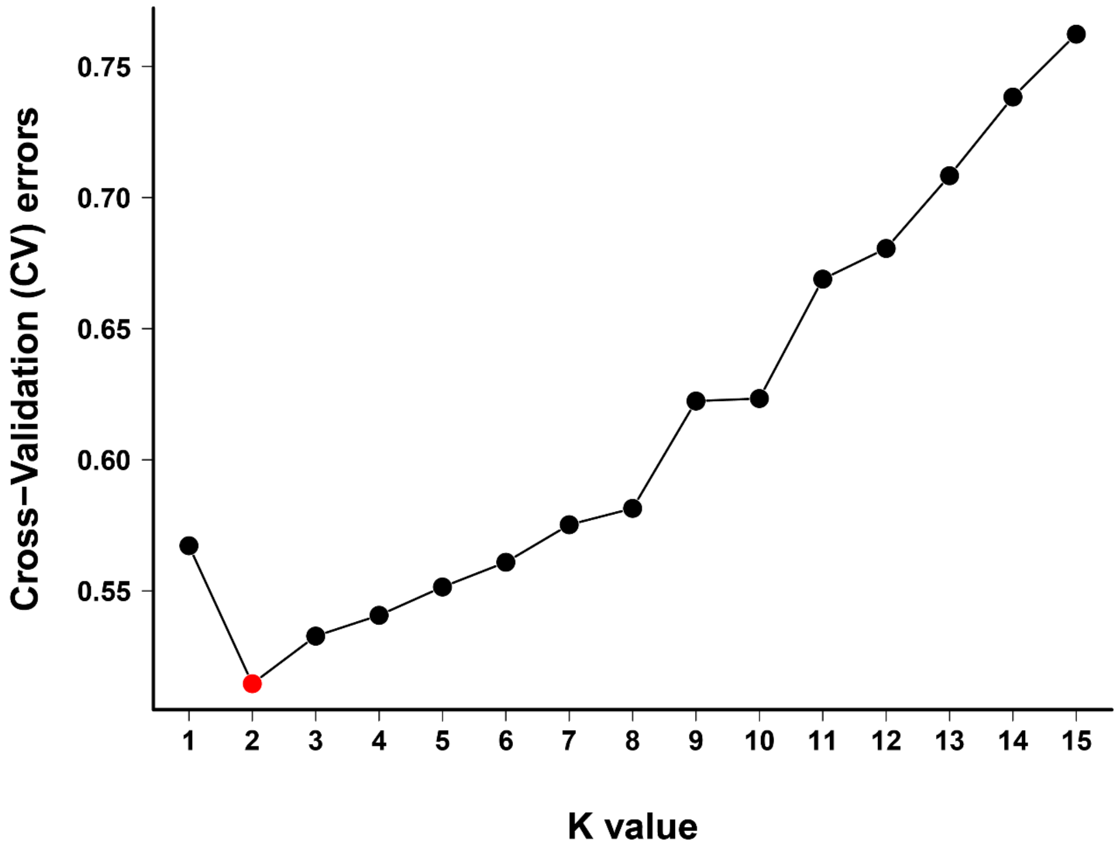

Figure S4 The Cross-Validation (CV) errors from 54 *Juglans nigra* and other 20 (sect. *Rhysocaryon*) species. Values at *K*=2, *K*=3 indicate two or three clusters as the most appropriate option.

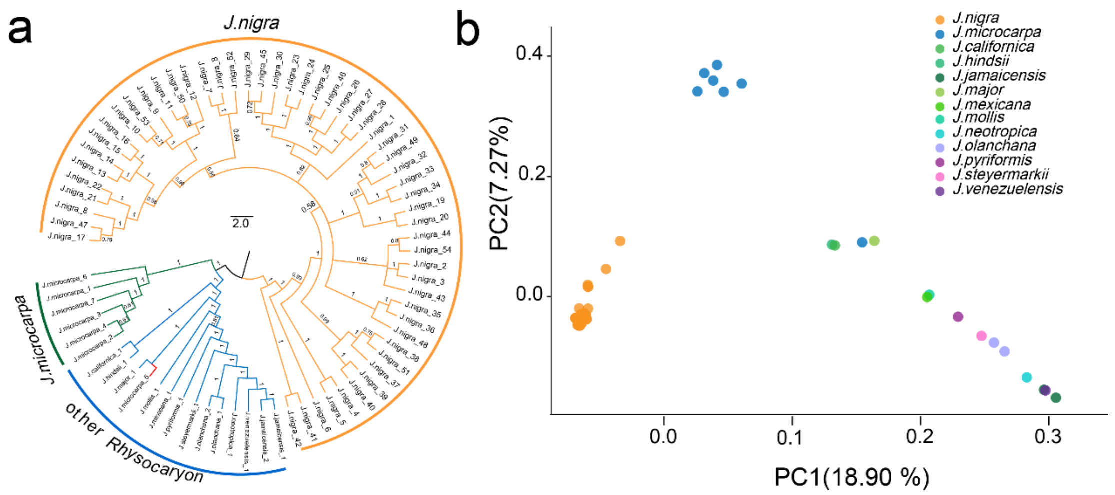

Figure S5 Phylogenetic relationships and PCA of *Juglans nigra*. (a) Neighbor-joining (NJ) phylogenetic tree constructed based on 92,560,670 whole-genome resequencing SNPs. (b) PCA plots of the first two components. *J. nigra* (yellow); *J. microcarpa* (blue), and all other samples.

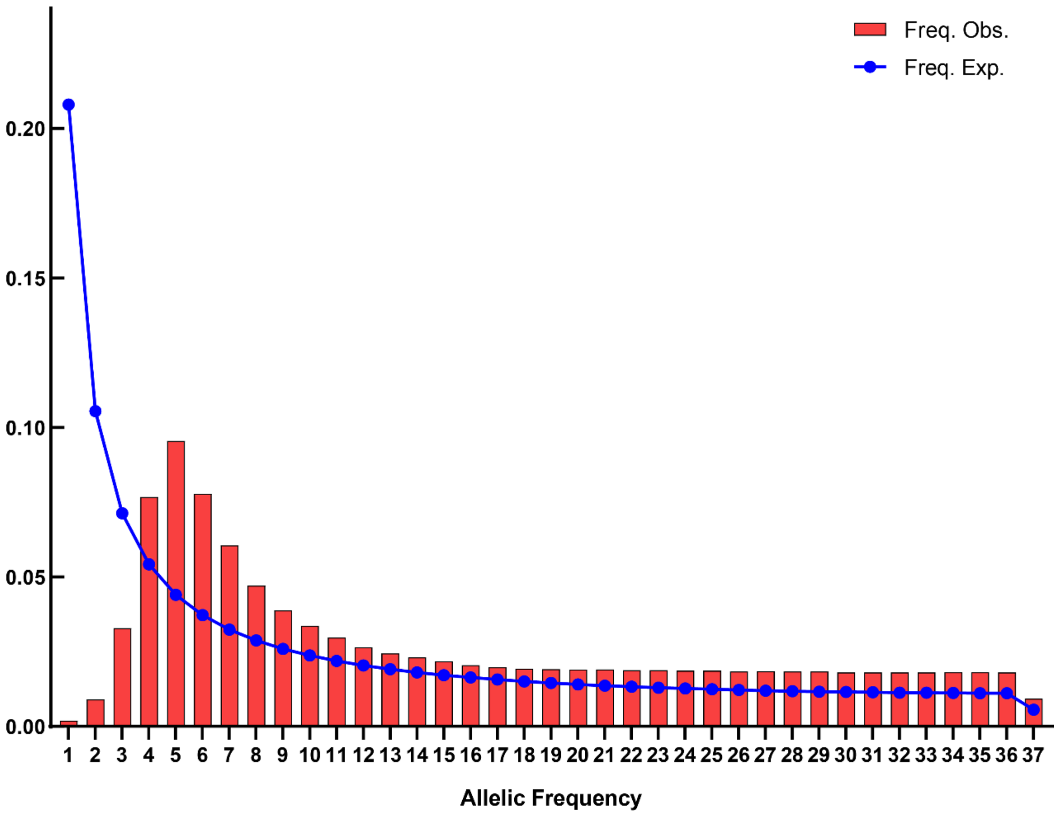

Figure S6 The mismatch distribution of *Juglans nigra* populations.

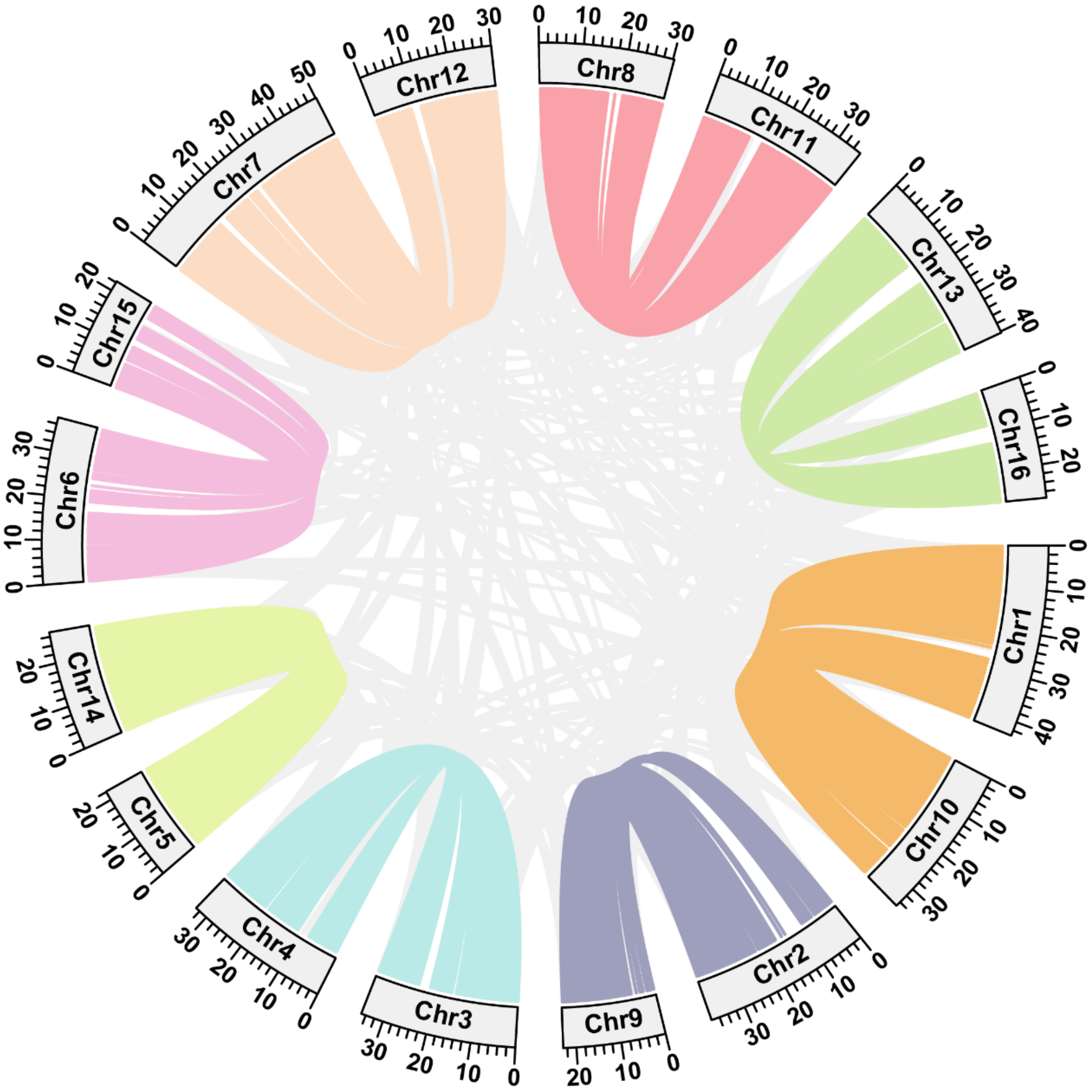

Figure S7 The paralogous syntenic relationships among sixteen chromosomes of *Juglans nigra*. The eight main duplication sub genomes, and the chromosome pairs as follows: Chr1 and Chr10, Chr2 and Chr9, Chr3 and Chr4, Chr5 and Chr14, Chr6 and Chr15, Chr7 and Chr12, Chr8 and Chr11, and Chr13 and Chr16.

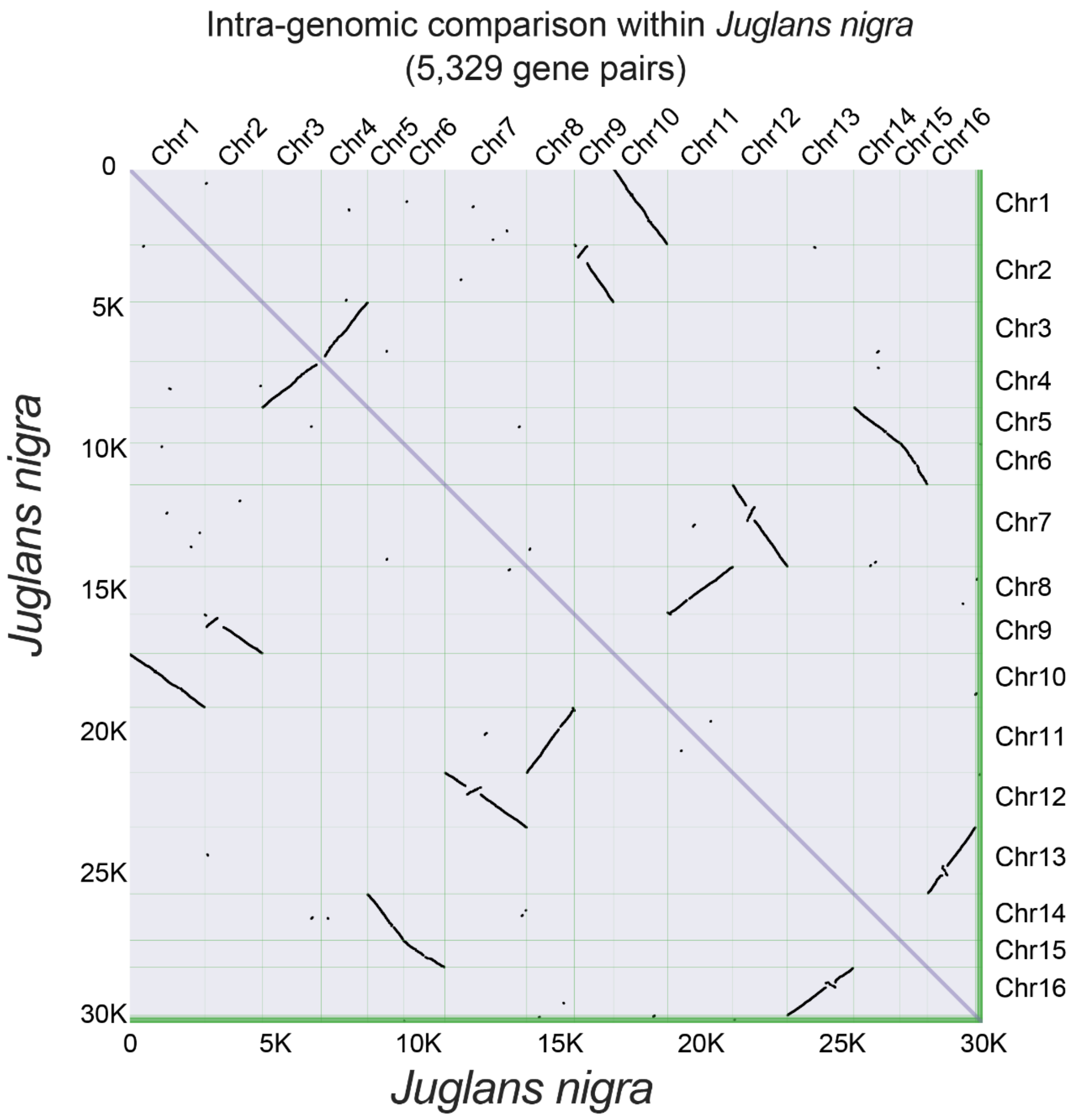

Figure S8 Dot-plot alignments within the assembled eastern black walnut (*Juglans nigra*) chromosomes. The black dots indicate homoeologous chromosomes within a genome. The dotted black line lines indicate paralogues produced by the whole-genome duplication event (WGD) and γ whole-genome triplication.

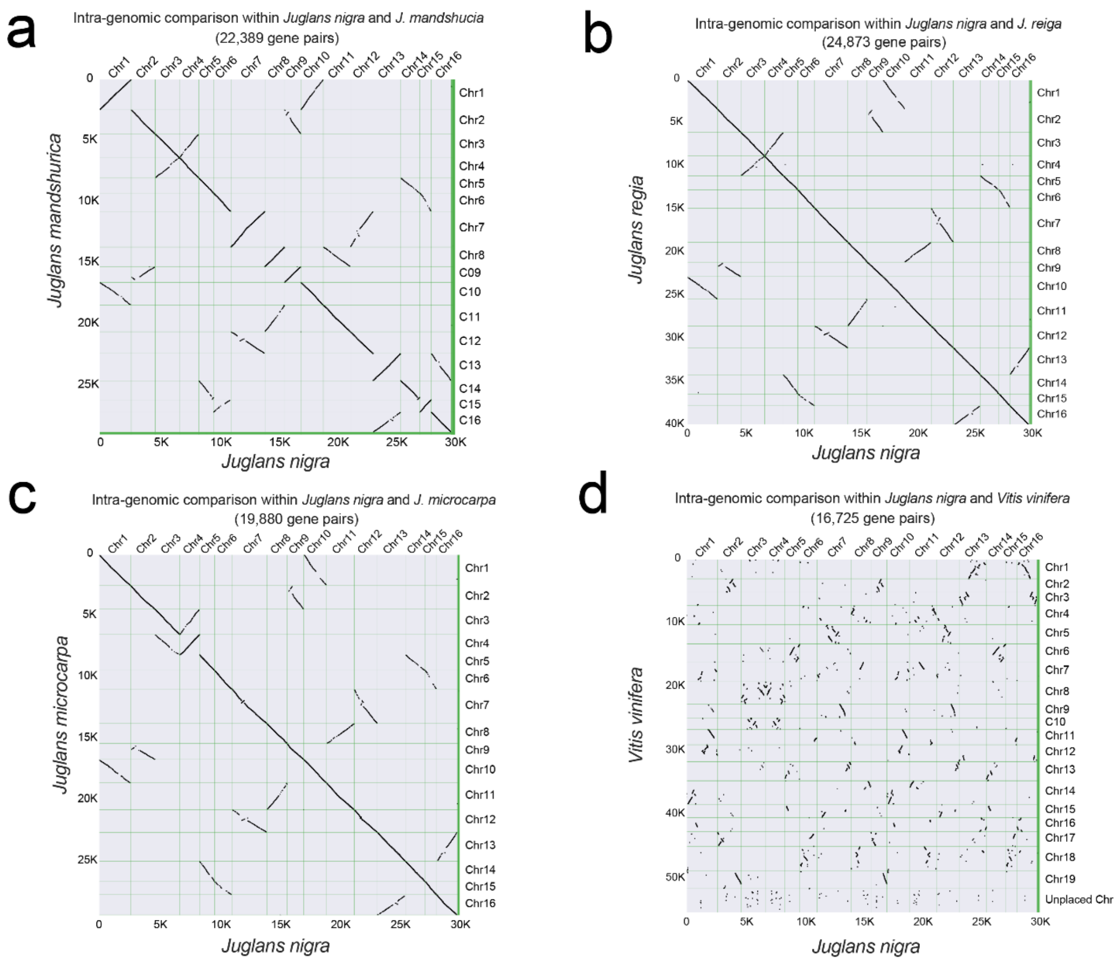

Figure S9 Dot-plot alignments of the assembled eastern black walnut (*Juglans nigra*) chromosomes and its relatives. (a) *J. nigra* chromosomes against *J. mandshurica* chromosomes. (b) *J. nigra* chromosomes against *J. regia* chromosomes. (c) *J. nigra* chromosomes against *J. microcarpa* chromosomes. (d) *J. nigra* chromosomes against *Vitis vinifera* chromosomes. The starts of the chromosomes are to the left or bottom. The black diagonal lines across the plots of the 16 chromosomes indicate interspecific collinearity of homoeologous chromosomes. The black dots indicate homoeologous chromosomes within a genome. The dotted black line lines indicate paralogues produced by the whole-genome duplication event (WGD) and the γ whole-genome triplication.

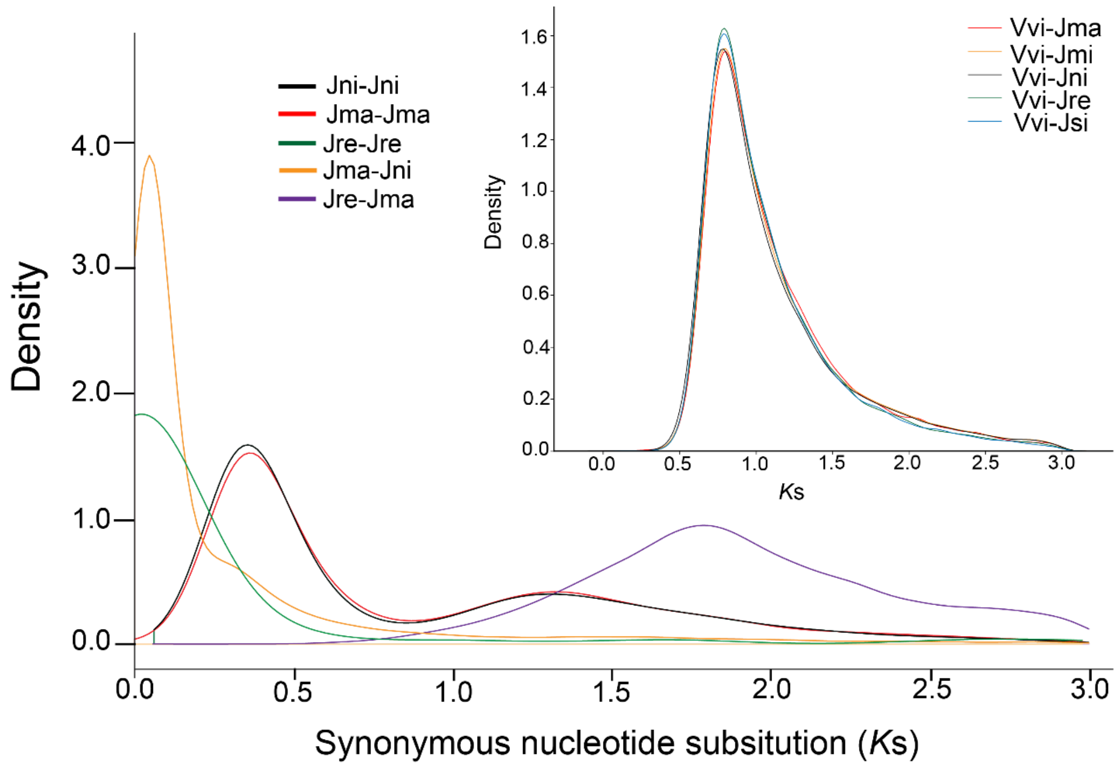

Figure S10 Whole-genome duplication and triplication in the *Juglans* lineages. Densities of Ks divergence in the BLASTP homology searches of five *Juglans* species (*J. nigra*, *J. mandshurica*, *J. microcarpa*, *J. regia*, and *J. sigillata*) against grape (*Vitis vinifera*) genome (upper right corner) and densities of Ks divergence of within *J. nigra*, within *J. mandshurica*, within *J. regia*, *J. nigra* against *J. mandshurica*, and *J. regia* against *J. mandshurica*.

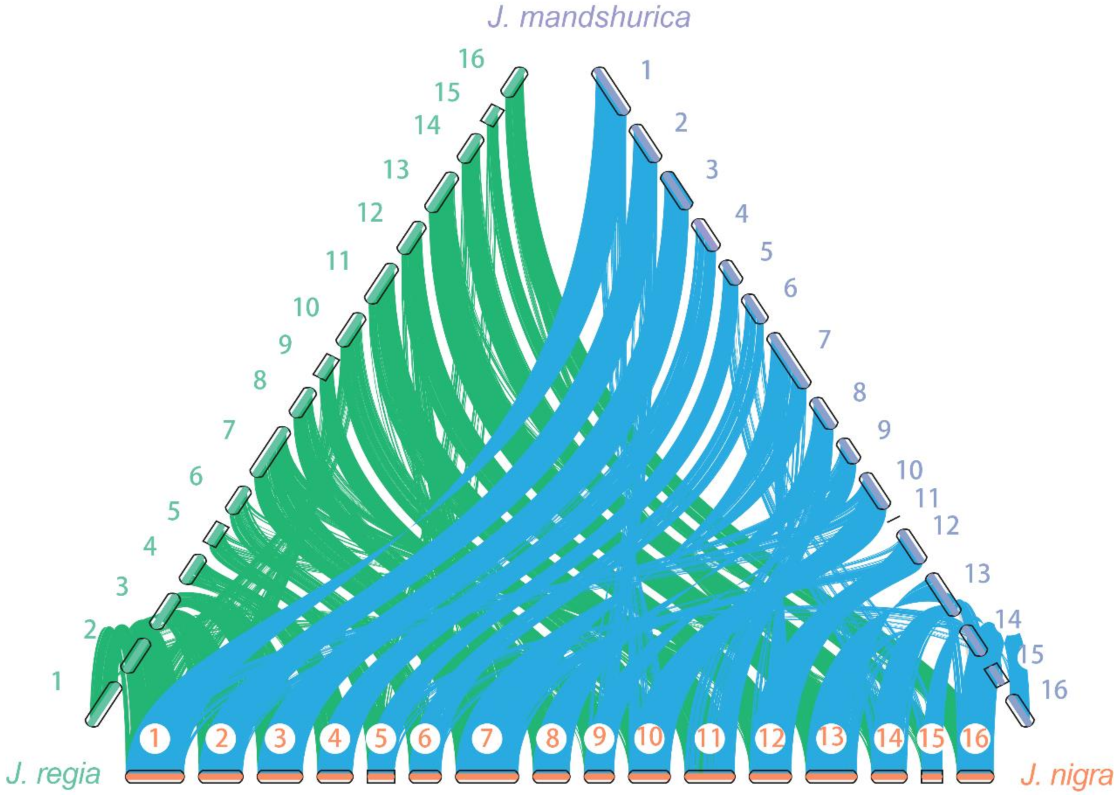

Figure S11 Syntenic analysis of eastern black walnut (*Juglans nigra*, orange), Persian walnut (*J. regia*, green), and *J. mandshurica* (light purple). Large areas of synteny are indicated by two colors. Green color indicates that the large areas of synteny between *J. nigra* and *J. regia*. Blue color indicates that the large areas of synteny between *J. nigra* and *J. mandshurica*.

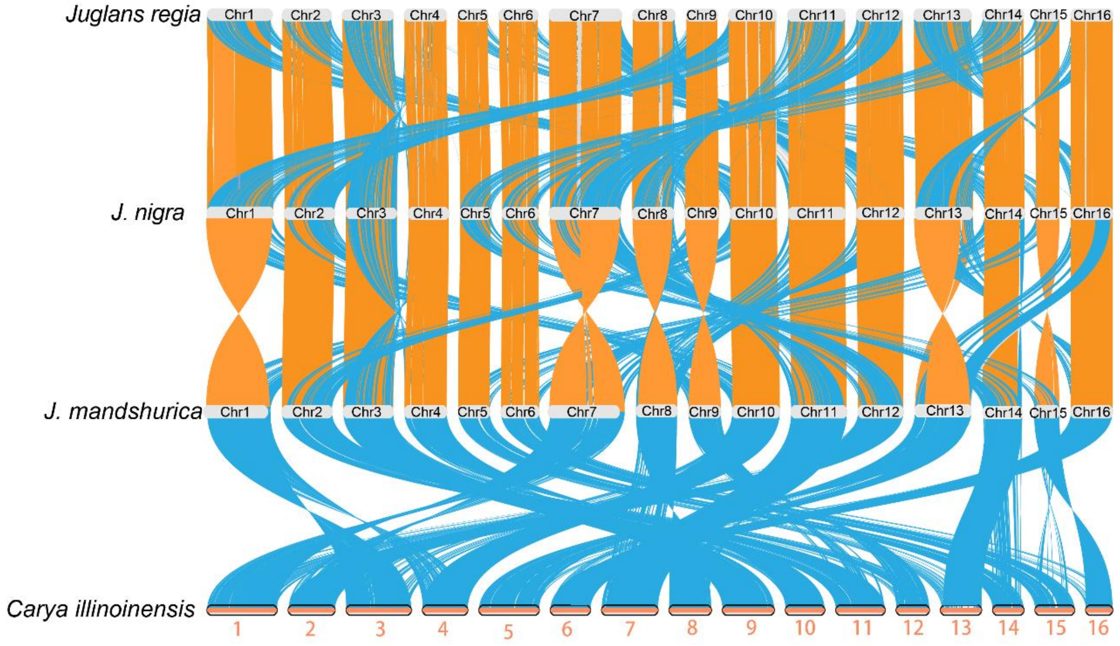

Figure S12 Syntenic analysis of eastern black walnut (*Juglans nigra*), Persian walnut (*J. regia*), *J. mandshurica* and *Carya illinoinensis* (pecan). The orange lines indicate high collinearity of homoeologous chromosomes across genomes. Blue lines indicate collinear inversions across genomes. The reference genomes were as follows: (*J. regia* (Marrano et al., 2020), *J. mandshurica* (Yan et al., 2021), and *Carya illinoinensis* (Huang et al., 2019).

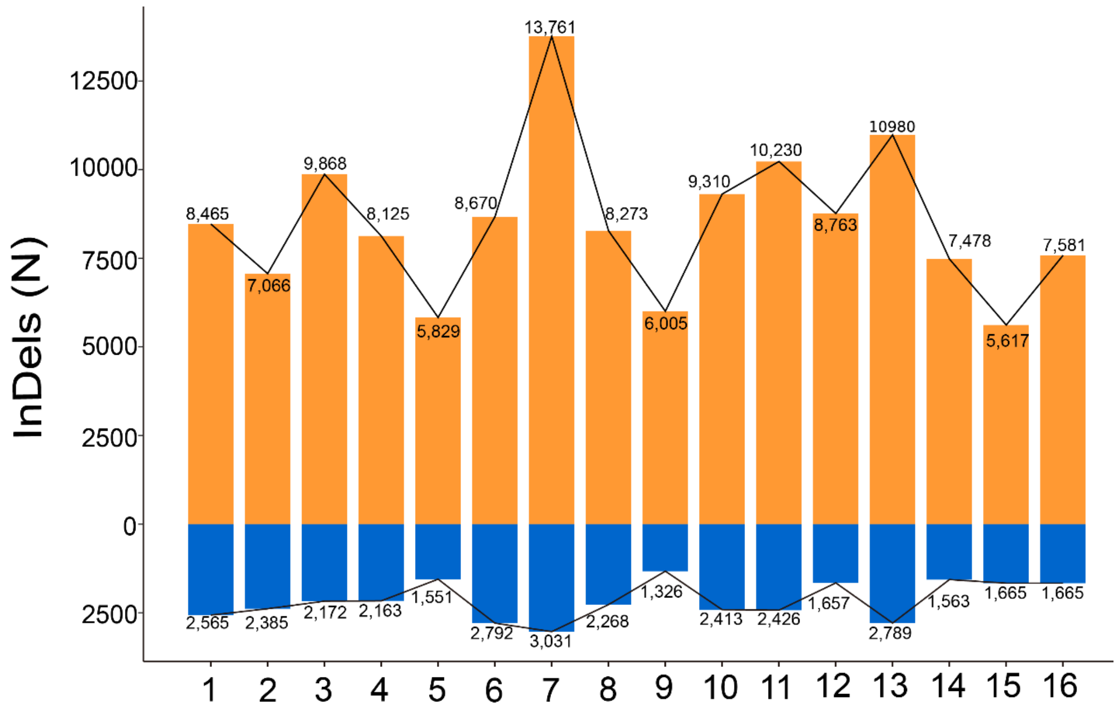

Figure S13 The number of InDels (deletions and insertions) and their chromosome distributions across the genomes of *J. nigra* vs. *J. regia* (yellow), and *J. nigra* vs. *J. microcarpa* (blue).

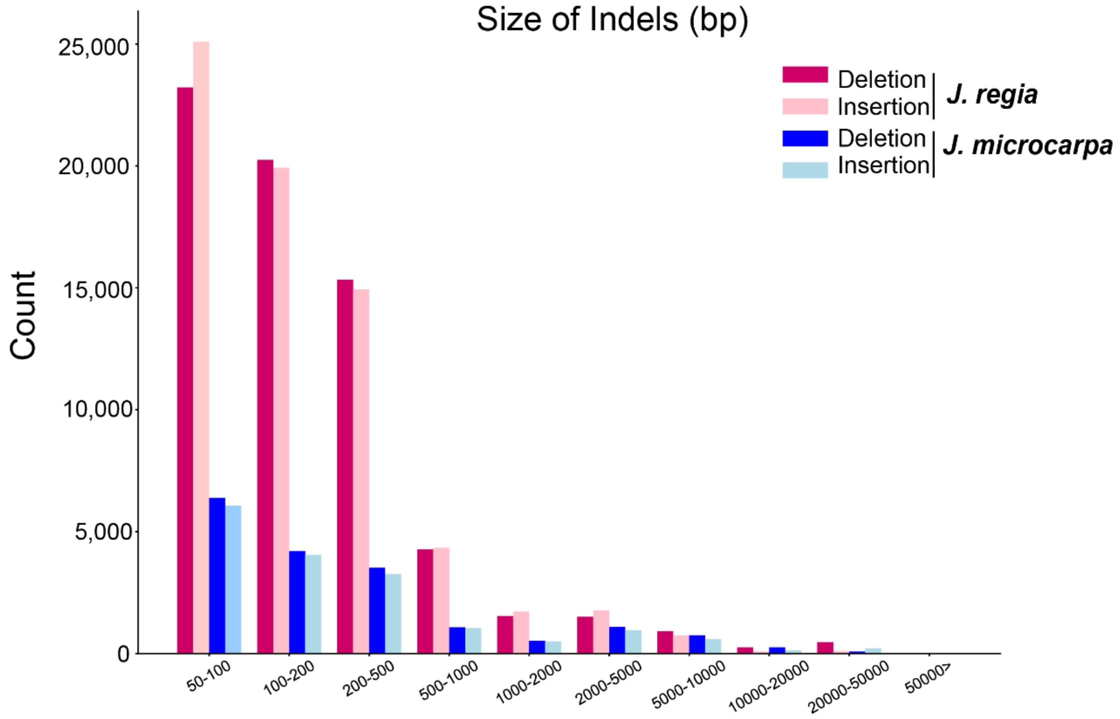

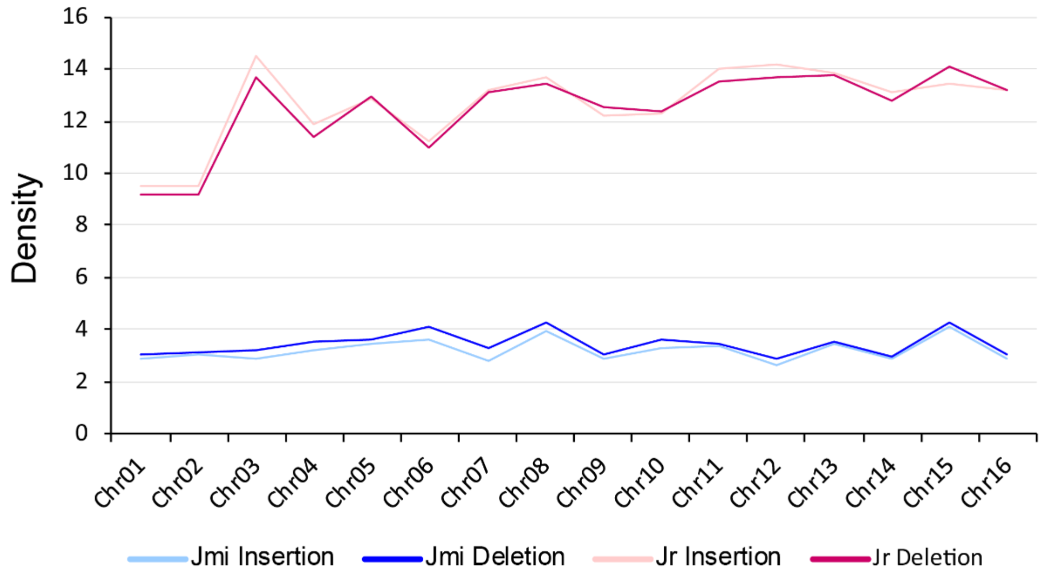

Figure S14 The InDels (deletions and insertions) density and distribution for each chromosome of *Juglans nigra* genome against the *J. regia* (Jr) and *J. microcarpa* (Jmi) assemblies.

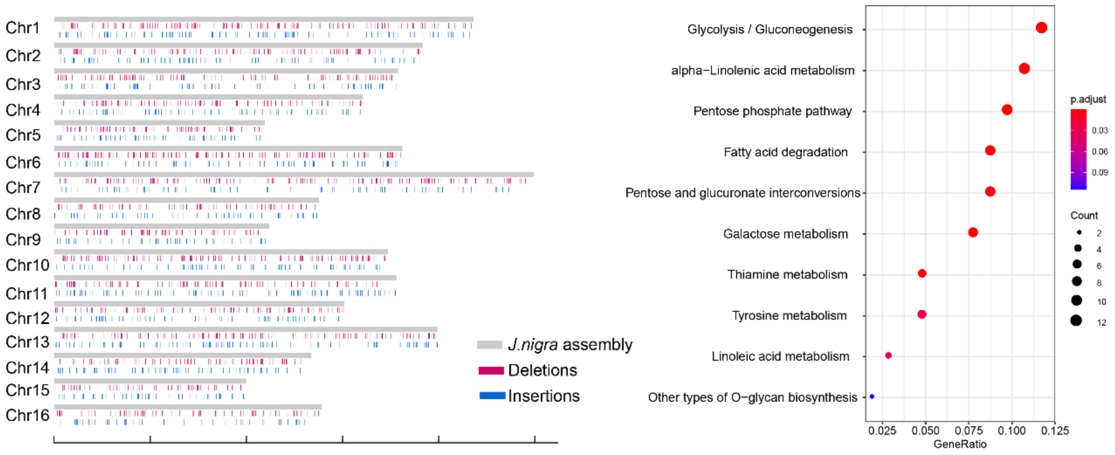

Figure S15 InDels distribution and the KEGG enrichment analysis of the genes in Indels>5 kb of the *Juglans nigra* assembly genome compared to the *J. microcarpa* genome.

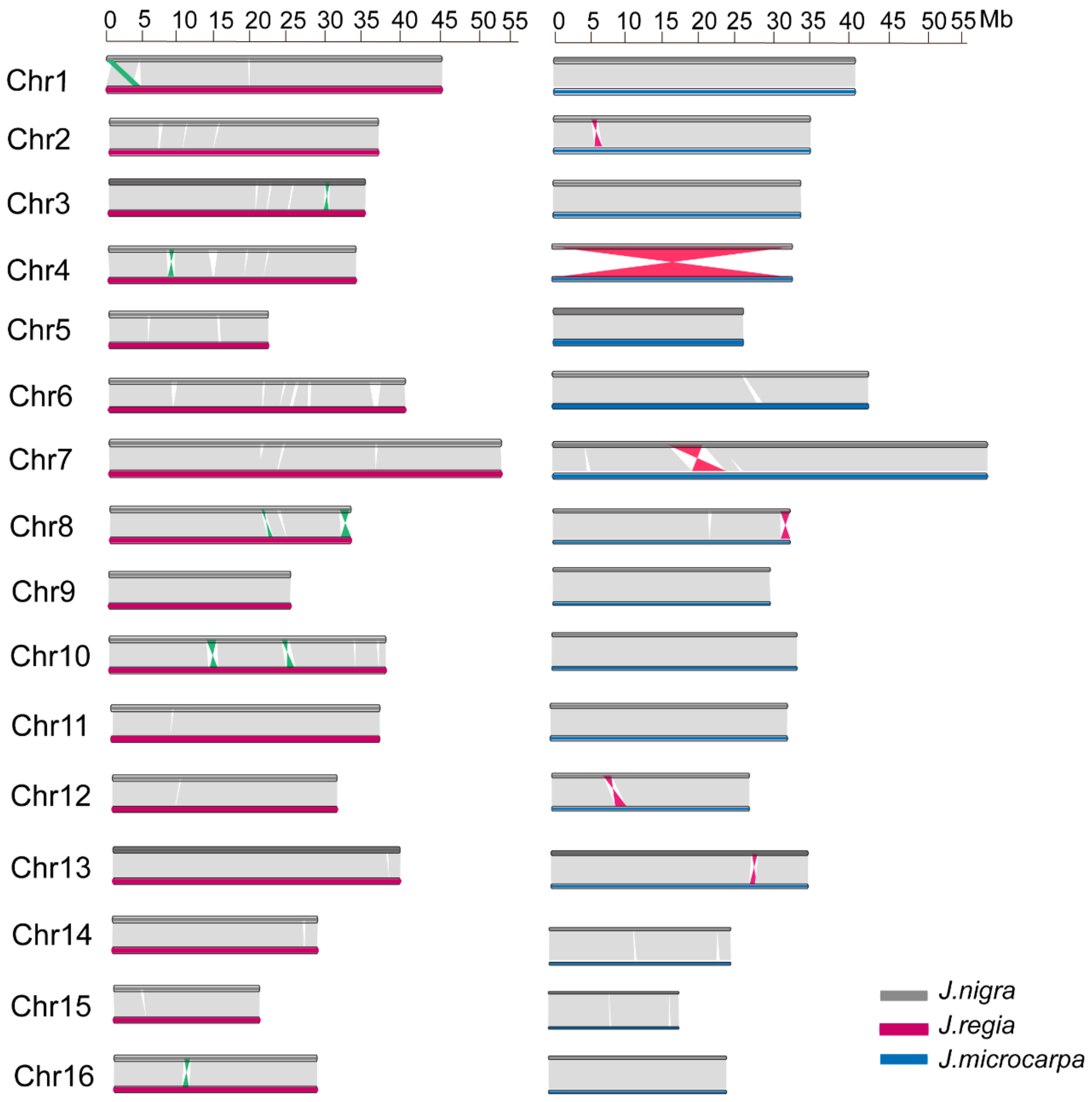

Figure S16 Genome collinearity between *Juglans nigra* and *J. regia* and *J. microcarpa*. Regions of collinearity smaller than 100 kb are filtered out. The green and purple lines indicate syntenic blocks more than 600 kb in length.

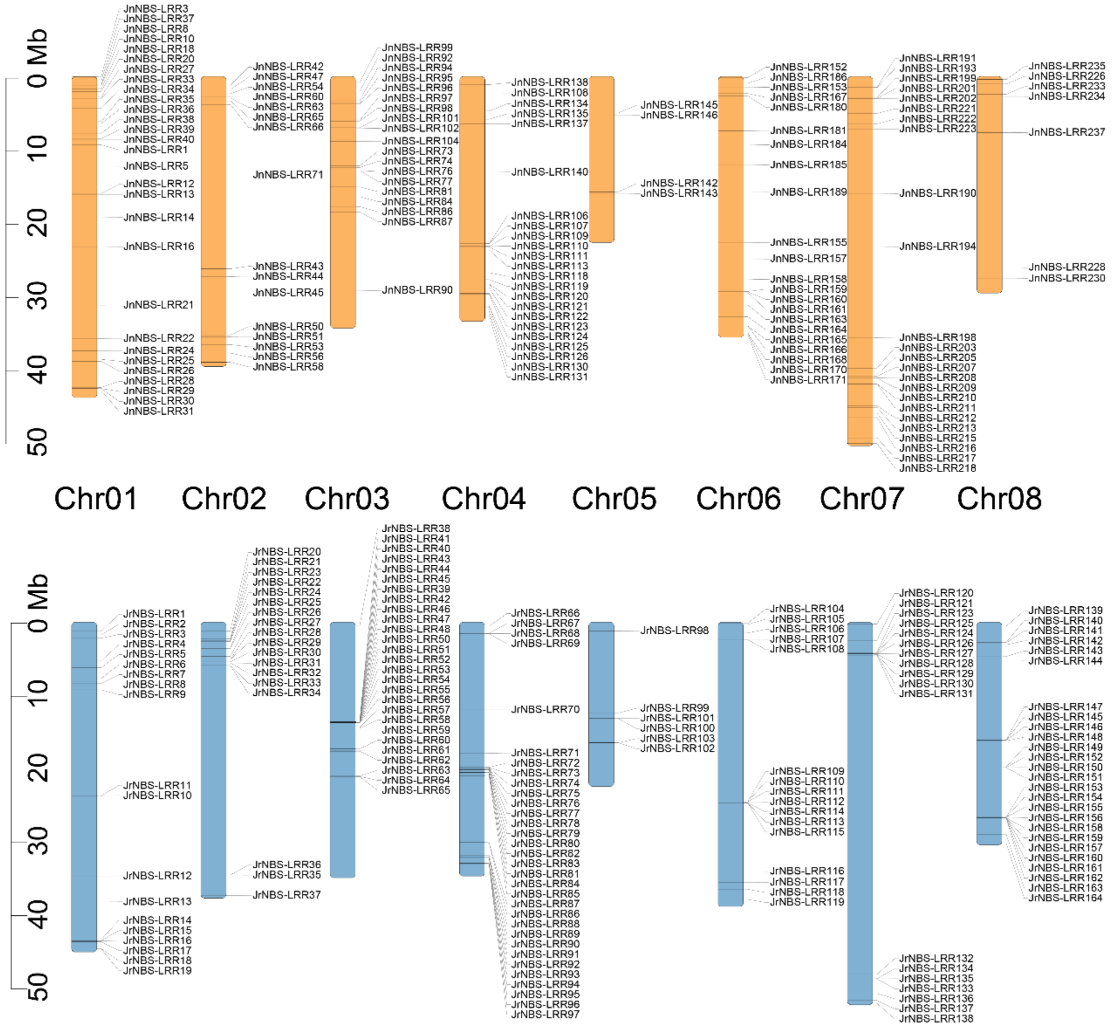

Figure S17 Chromosomal (1-8) distribution of *NBS-LRR* genes in *Juglans nigra* and *J. regia*.

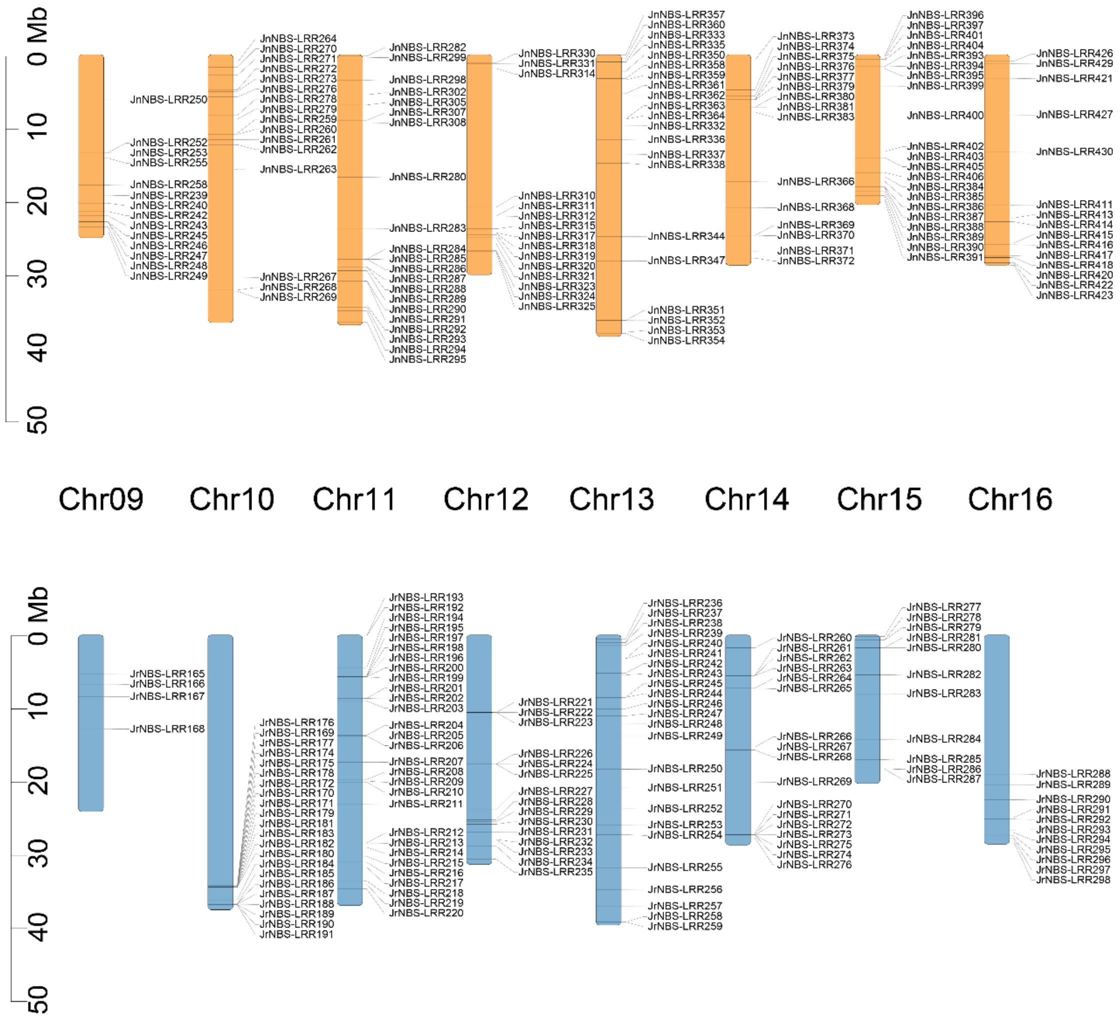

Figure S18 Chromosomal (9-16) distribution of *NBS-LRR* genes in *Juglans nigra* and *J. regia*.

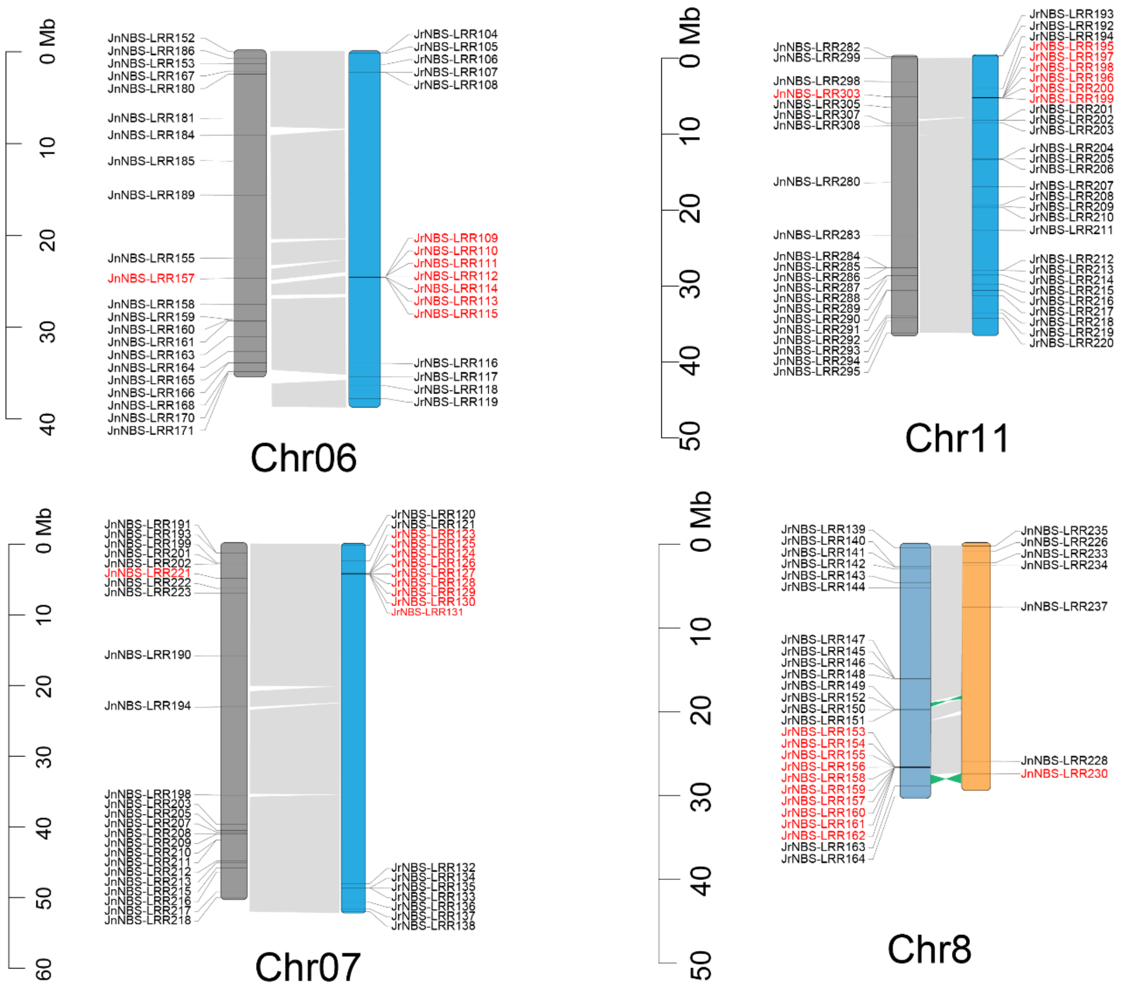

Figure S19 Distributions of a single *NBS-LRR* of *J. nigra* and gene clusters on *J. regia* chromosomes 6, 7, 8, and 1.

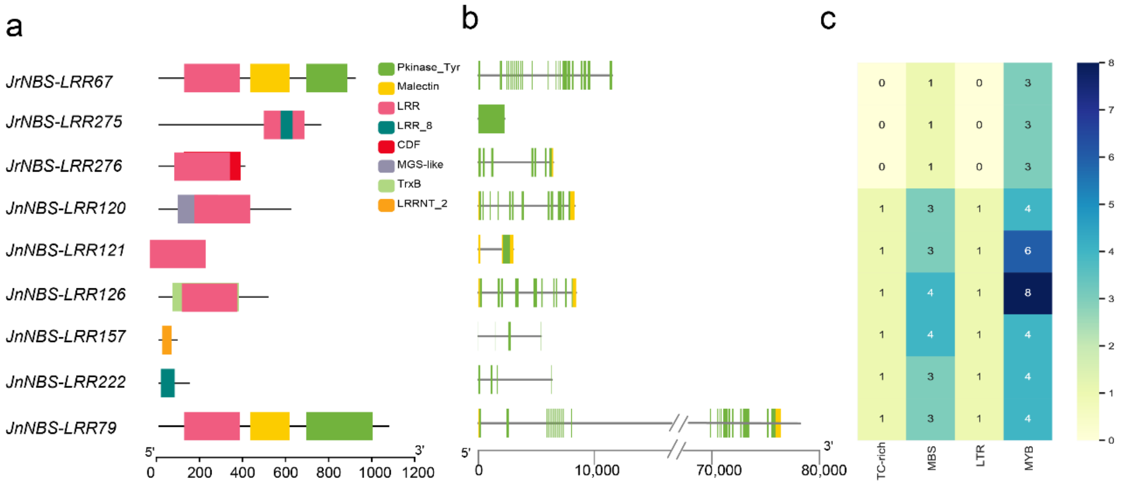

Figure S20 Clustering of *NBS-LRR* genes, their structure, domains, and Cis-acting elements in promoter regions. (a) Protein domains of nine *NBS-LRR* genes of *J. nigra* and *J. regia*. (b) Gene structure of nine *NBS-LRR* genes of *J. nigra* and *J. regia*. (c) Cis-acting elements in promoter regions of nine *NBS-LRR* genes of *J. nigra* and *J. regia*.

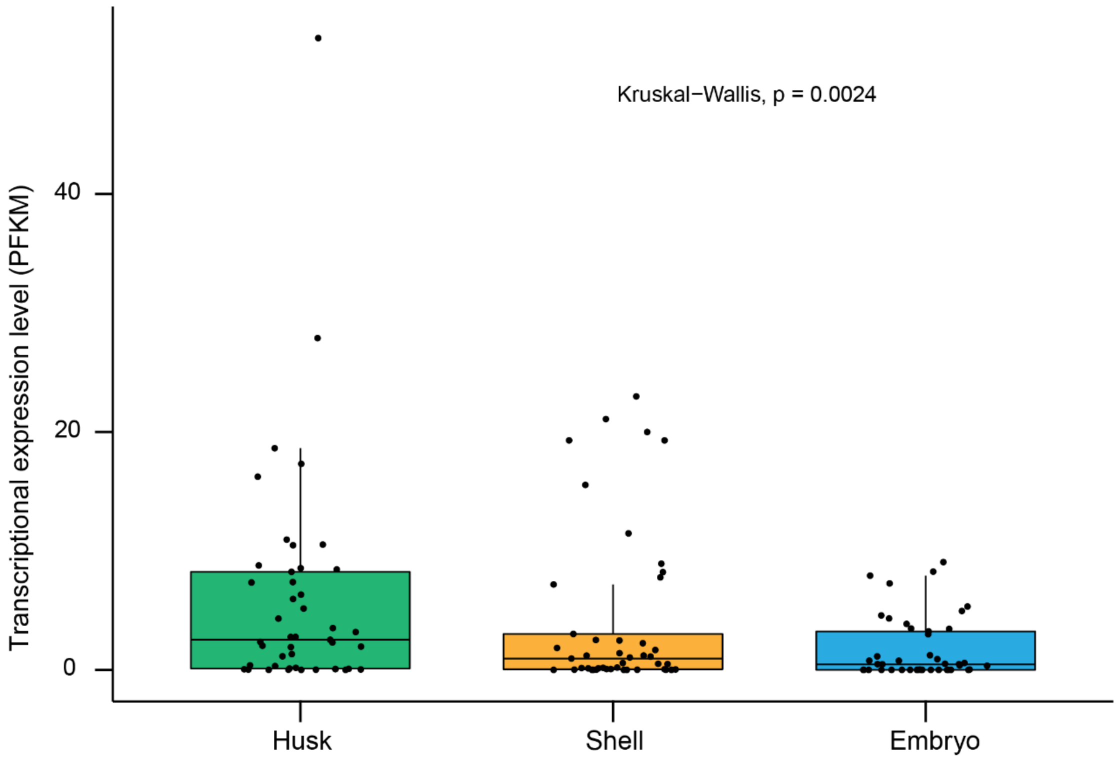

Figure S21 Result of transcriptome expression levels of five candidate *NBS-LRR* genes (*JnNBS-LRR126*, *JnNBS-LRR80*, *JnNBS-LRR157*, *JnNBS-LRR1221*, and *JnNBS-LRR303*) of husks, shell, and embryo in *Juglans nigra*. (H=husk; S=shell; E= embryo). Each tissue including three development stages (80 DAF, 111 DAF, and 140 DAF).
